# Deconvolution of bulk blood eQTL effects into immune cell subpopulations

**DOI:** 10.1186/s12859-020-03576-5

**Published:** 2020-06-12

**Authors:** Raúl Aguirre-Gamboa, Niek de Klein, Jennifer di Tommaso, Annique Claringbould, Monique GP van der Wijst, Dylan de Vries, Harm Brugge, Roy Oelen, Urmo Võsa, Maria M. Zorro, Xiaojin Chu, Olivier B. Bakker, Zuzanna Borek, Isis Ricaño-Ponce, Patrick Deelen, Cheng-Jiang Xu, Morris Swertz, Iris Jonkers, Sebo Withoff, Irma Joosten, Serena Sanna, Vinod Kumar, Hans J. P. M. Koenen, Leo A. B. Joosten, Mihai G. Netea, Cisca Wijmenga, BIOS Consortium, Lude Franke, Yang Li

**Affiliations:** 1 Department of Genetics, University of Groningen, University Medical Center Groningen, Groningen, the Netherlands; 2 Department of Genetics, Oncode Institute, University of Groningen, University Medical Center Groningen, Groningen, the Netherlands; 3 Estonian Genome Centre, Institute of Genomics, University of Tartu, Tartu, Estonia; 4 Centre for Individualised Infection Medicine (CiiM) & TWINCORE, joint ventures between the Helmholtz-Centre for Infection Research (HZI) and the Hannover Medical School (MHH), Feodor-Lynen-Str. 7, 30625 Hannover, Germany; 5 University of Groningen and University Medical Center Groningen, Genomics Coordination Center, Groningen, the Netherlands; 6 Department of Laboratory Medicine, Laboratory for Medical Immunology, Radboud University Medical Centre, Nijmegen, the Netherlands; 7 Department of Internal Medicine and Radboud Center for Infectious Diseases, Radboud University Medical Center, Nijmegen, the Netherlands; 8 Department of Genomics & Immunoregulation, Life and Medical Sciences Institute (LIMES), University of Bonn, Bonn, Germany

**Keywords:** eQTL, Deconvolution, Cell types, Immune cells

## Abstract

**Background:**

Expression quantitative trait loci (eQTL) studies are used to interpret the function of disease-associated genetic risk factors. To date, most eQTL analyses have been conducted in bulk tissues, such as whole blood and tissue biopsies, which are likely to mask the cell type-context of the eQTL regulatory effects. Although this context can be investigated by generating transcriptional profiles from purified cell subpopulations, current methods to do this are labor-intensive and expensive. We introduce a new method, *Decon2,* as a framework for estimating cell proportions using expression profiles from bulk blood samples (Decon-cell) followed by deconvolution of cell type eQTLs (Decon-eQTL).

**Results:**

The estimated cell proportions from Decon-cell agree with experimental measurements across cohorts (R ≥ 0.77). Using Decon-cell, we could predict the proportions of 34 circulating cell types for 3194 samples from a population-based cohort. Next, we identified 16,362 whole-blood eQTLs and deconvoluted cell type interaction (CTi) eQTLs using the predicted cell proportions from Decon-cell. CTi eQTLs show excellent allelic directional concordance with eQTL (≥ 96–100%) and chromatin mark QTL (≥87–92%) studies that used either purified cell subpopulations or single-cell RNA-seq, outperforming the conventional interaction effect.

**Conclusions:**

Decon2 provides a method to detect cell type interaction effects from bulk blood eQTLs that is useful for pinpointing the most relevant cell type for a given complex disease. Decon2 is available as an R package and Java application (https://github.com/molgenis/systemsgenetics/tree/master/Decon2) and as a web tool (www.molgenis.org/deconvolution).

## Background

For many of the genetic risk factors that have been associated to immune diseases by genome-wide association studies (GWAS), the molecular mechanism leading to disease remains unknown [1]. Most of these genetic risk variants are located in the non-coding regions of the genome, implying that they play a role in gene regulation [2, 3]. Expression quantitative trait locus (eQTL) analysis provides a way to characterize the regulatory effect of these risk factors in humans, and many eQTL studies have now been carried out using bulk tissues, for example, whole blood [4, 5]. However, bulk tissues comprise many different cell types, and gene regulation is known to vary across cell types [6–8]. In recent years, efforts to describe eQTL effects in purified cell subpopulations have been carried out in specific cell types [9]. Unfortunately, the length and cost of the study protocols have limited these studies to small sample sizes and only a few cell types. Current developments on single cell (sc) RNASeq technologies have given rise to sc-eQTLs, an approach that, although promising, is still bound to a limited number of individuals, which thereby limits the number of detectable cell type interaction (CTi) eQTLs. Nevertheless, the ability to pinpoint the CT in which a risk factor exerts an eQTL effect could help us to understand its role in disease.

Statistical approaches to detect CT effects using tissue expression profiles have mainly been developed to evaluate gene by environment interaction (GxE) terms, for example to detect CT eQTLs for myeloid and lymphoid lineages using only whole blood gene expression and by evaluating the interaction between genotype and cell proportions for neutrophils and lymphocytes in whole blood [10]. A second study linked eQTL genes to proxy genes through correlation; these proxy genes were then associated with intrinsic or extrinsic factors such as cell proportions or inflammation markers [11]. However, these efforts focused on exploiting only one GxE term, or on indirectly linking the CT proportions to given eQTL, rather than directly ascertaining the interaction between all the main cell proportions comprising the bulk tissue and genotype. Unfortunately, quantifying cell proportions, in particular rare subpopulations (total abundance ≤3% in circulating white blood cells), is expensive and time-consuming. Hence, quantifying immune cell proportions in large functional genomics cohorts is not common practice.

Here we present and validate Decon2, a computational and statistical framework that can (1) predict the proportions of known circulating immune cell subpopulations (Decon-cell), and (2) combine these predicted proportions with whole blood gene expression and genotype information to assign bulk eQTL effects into CTi eQTLs (Decon-eQTL). Our two-step framework provides an improvement over previously published methods. Unlike earlier methods [12], Decon-cell does not rely on any prior information about transcriptome profiles from purified cell subpopulations. It only requires quantification of the cell proportions comprising the bulk tissue, in this case whole blood. Decon-cell identifies signature genes that correlate with cell proportions in a bulk tissue. Secondly, Decon-eQTL is the first approach in which all major cell proportions (the major cell types for which the sum of proportions per sample is approximately 100%) of bulk blood tissue are incorporated into an eQTL model simultaneously. Decon-eQTL can then be used to systematically test for any significant interaction between each CT and genotype, while also controlling for the effect on expression of the other cell types.

We generated the Decon-cell predictive models using data from the 500FG cohort [13], where quantification of immune cell types was carried out using FACS [14] and RNA-Seq-based bulk whole blood transcriptome profiles were available for 89 samples [15]. By using a cross-validation approach, we were able to accurately predict 34 out of 73 cell subtypes using only whole blood gene expression. For validation, we applied Decon-cell to three independent cohorts (Lifelines Deep [16], *n* = 627; Leiden Longevity cohort [17], *n* = 660 and the Rotterdam Study [18], *n* = 773) for which both blood RNA-seq and measured cell proportion data are available (neutrophils, lymphocytes and CD14+ monocytes and granulocytes). Additionally, we benchmarked Decon-cell prediction performance against two other existing methods that quantify immune cell composition using gene expression profiles from whole blood on these three independent cohorts. After showing that we can accurately predict circulating immune cell proportions, we applied Decon-cell to estimate cell proportions in 3194 individuals from the BIOS cohort [16, 19–21] for whom both whole blood RNA-seq and genotypes were available. The BIOS cohort is a valuable resource for functional genomics studies where extensive characterization of the genetic component on gene expression [11] and epigenetics [22] have been performed. We integrated whole blood expression and genotype information and predicted cell proportion with Decon-eQTL to deconvolute 16,362 significant whole blood *cis*-eQTLs top effects into CT interacting eQTLs (CTi eQTLs). These deconvoluted CTi eQTL results were comprehensively validated using transcriptome profiles from purified cell subpopulations [23], eQTLs and chromatin mark QTLs from purified cell types [9] and eQTLs from single-cell experiments [24]. We also systematically compared the performance of Decon-eQTL against the most used method [10] that detect cell type eQTL effects using whole blood expression profiles.

## Results

### Decon-cell accurately predicts the proportions of known immune cell types

**Fig. 1:**
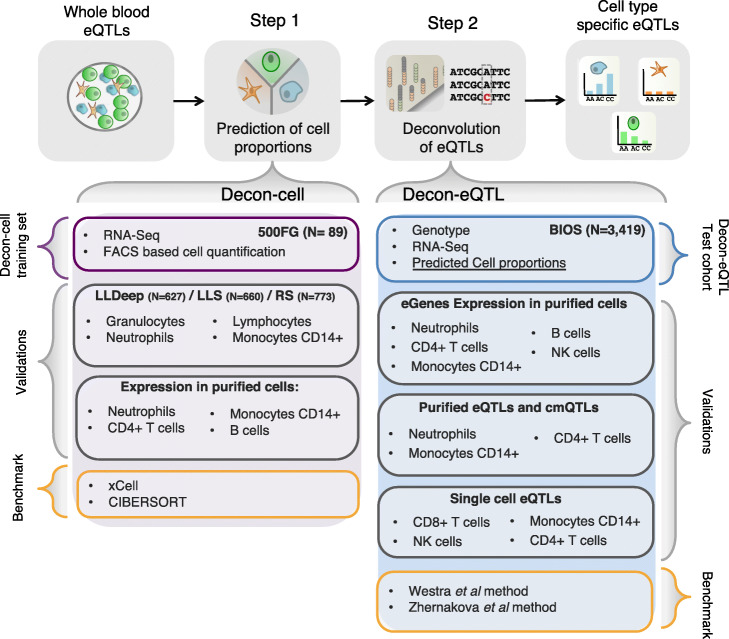
Workflow of application of Decon2 to predict cell counts followed by deconvolution of whole blood eQTLs. Using whole blood expression and FACS data of 500FG samples, Decon-cell predicts cell proportions with selected marker genes of circulating immune cell subpopulations. Validations of Decon-cell were carried out on three independent cohorts for which measurements of neutrophils/granulocytes, lymphocytes and monocytes CD14+ were available along with expression profiles of whole blood. Benchmarking of Decon-cell was performed against CIBERSORT [25] and xCell [12]. Decon-cell was applied to an independent cohort (BIOS) to predict cell counts using whole blood RNA-seq. Decon-eQTL subsequently integrates genotype and tissue expression data together with predicted cell proportions for samples in BIOS to detect cell type eQTLs. We validated Decon-eQTL using multiple independent sources, including expression profiles of purified cell subpopulations, eQTLs and chromatin mark QTLs (cmQTLs) from purified neutrophils, monocytes CD14+ and CD4+ T cells [9], and single-cell eQTL results [24]. Benchmarking of Decon-eQTL was carried out for comparison with a previously reported methods that detected cell type–eQTL effects using whole blood expression data, i.e. the Westra et al. [10]

In order to assign the cell types from which an overall eQTL effect from a bulk tissue sample (e.g. whole blood) arise, we need three types of information: genotype data, tissue expression data and cell type proportions (Fig. 1). Here we propose a computational method that predicts the cell proportions of known immune cell types using gene signatures in whole blood expression data using a machine-learning approach. Decon-cell employs the regularized regression method elastic net [26] to define sets of signature genes for each cell type. In other words, these signatures were selected as having the best prediction power for individual cell proportions.

**Fig. 2:**
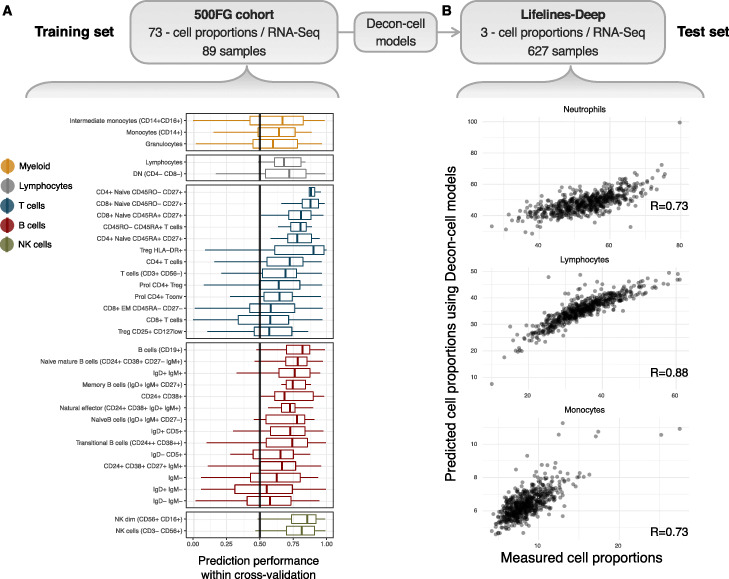
Prediction of cell proportions using whole blood transcriptome by Decon-cell. **a** Distribution of prediction performance (Spearman correlation coefficient) of the 34 predictable cell types in 100 iterations of prediction within the 500FG cohort. **b** Cross- cohort validation in an independent Lifelines-Deep cohort (*n* = 627): the measured and predicted cell proportions for neutrophils (given by granulocytes in 500FG), lymphocytes and monocytes are compared

There are 89 samples in the 500FG cohort with both whole blood RNA-seq and quantification of 73 immune cell subpopulations by FACS. This data was used to build the prediction models for estimating cell subpopulations by Decon-cell. First, we determined which of the 73 cell subpopulations could be reliably predicted by Decon-cell. A within-cohort cross-validation strategy was employed by randomly dividing 89 samples (Fig. 1) into training and test sets (70 and 30% of the samples, respectively). After generating a model using each training set, we applied the prediction models of each cell type to the samples in the test sets. We compared the predicted and measured cell proportion for each cell type using Spearman correlation coefficients to evaluate the prediction performance. We repeated this process 100 times and then used the mean of the correlation coefficient in all 100 iterations to evaluate the prediction performance. We were able to predict 34 out of 73 cell subpopulations using whole blood gene expression data at a threshold of mean R ≥ 0.5 across all 100 iterations (Fig. 2a, Supplementary Fig. 1, Supplementary Table 1). The number of signature genes selected in our models for predicting cell proportions varied across the cell types, ranging from 2 to 217 signature genes (Supplementary Fig. 2A, Supplementary Table 1), and they were independent of the average abundance of these cell types in whole blood (R = 0.02, Spearman correlation coefficient, Supplementary Fig. 2A). In particular, cell types that are abundant in whole blood (granulocytes-neutrophils, CD4+ T-cells and CD14+ monocytes) were predicted with high confidence (correlation between predicted and measured values, R ≥ 0.73). Remarkably, we were also able to predict a number of less abundant cell subpopulations, including NK cells, CD8+ T-cells, non-NK T-cells (CD3- CD56-), CD4+ central memory, CD4+ effector memory T-cells and regulatory T-cells (Supplementary Fig. 2A), as determined by FACS. Cell types with a low prediction performance (R < 0.5) are those that have few signature genes with expression levels that correlate sufficiently (i.e. absolute R < 0.3) with the measured cell proportions in whole blood (Supplementary Fig. 2B-C). For each of the 34 predictable cell types, we used Decon-cell to build models for predicting their cell counts using all 89 samples from the 500FG cohort. These models were applied to 3194 samples in an independent cohort (BIOS cohort) to predict cell proportions of circulating immune cell types for the subsequent deconvolution of eQTL effects.

In addition to within-cohort validation, we tested our cell proportion models using three independent cohorts (LLDeep, *n* = 627; LLS, *n* = 660; RS, *n* = 773) in which cell type abundances were quantified using a Coulter counter for neutrophils (granulocytes for RS), lymphocytes and CD14+ monocytes (Fig. 2b, Supplementary Fig. 3A-B). In LLDeep, we were able to accurately predict these three cell types with Spearman correlation coefficients of R = 0.73, R = 0.89 and R = 0.73, respectively. For LLS and RS, the prediction performance was similarly accurate for neutrophils and lymphocytes (R = 0.76 for neutrophils, R = 0.84 for lymphocytes), but less so for monocytes (R = 0.50 for CD14+ monocytes and proportions in LLS and R = 0.74 for granulocytes, R = 0.83 for lymphocytes and R = 0.28 for CD14+ monocytes in RS).

Next, in order to benchmark Decon-cell, we compared its prediction performance against two other existing tools that quantify the abundance of known immune cell types using bulk whole blood expression profiles: CIBERSORT [25] and xCell [12]. We obtained the predicted proportions by CIBERSORT and enrichment scores of circulating immune cells by xCell for the samples in three different cohorts: LLDeep, LLS and RS (Supplementary Fig. 4A-B). For each cell type, Decon-cell outperforms CIBERSORT and xCell (Supplementary Fig. 3B). The scatterplots of predicted vs measured values (Supplementary Fig. 3 A, Supplementary Fig. 4 A-B) further demonstrate that the better performance of Decon-cell is not due to cell proportion outliers.

Finally, we evaluated whether the signature genes showed CT expression in their relevant purified cell types using BLUEPRINT [23] RNA-seq data from the purified cell subpopulations. Here we focused on cell types with more than three samples measured, which included neutrophils, CD14+ monocytes, CD4+ T-cells and B-cells. The signature genes showed overall higher expression in their relevant cell subpopulations compared to other cell subpopulations. Interestingly, the signature genes were also able to cluster the samples of the relevant CT using unsupervised hierarchical clustering (Supplementary Fig. 5A-D). Together, our results demonstrate that the gene signatures identified by Decon-cell using only whole blood gene expression data are predictive for the proportions of circulating immune cell subpopulations.

To facilitate the cell proportion prediction of new samples using whole blood RNA-seq, we have made the Decon-cell prediction models and gene signatures available in an R package (Decon-cell) and as a web tool (www.molgenis.org/deconvolution). These two implementations allow users to pre-process their RNA-seq expression counts and estimate cell proportions using the pre-established models for 34 cell types in whole blood. In addition, the Decon-cell R package allows users to generate Decon-cell-like gene signatures to predict their own cell proportions, which requires the input of bulk expression profiles and cell proportions to generate new Decon-cell predictive models.

### Decon-eQTL identifies which cell types contribute to the whole blood eQTL effect

As we know, eQTL analysis using whole blood bulk expression data fails to distinguish between a general eQTL present in all cell types and an effect mainly found in a subset of the cell types. We therefore propose a new approach, called Decon-eQTL, that assigns the overall bulk eQTL into CT effects. Using the cell proportions in whole blood, it is possible to formally test if the genetic effect is interacting with the cell proportions. More explicitly, we include both the genotype and all major CT proportions of interest in a linear model, and systematically test if there is a significant interaction effect between genotype and each of the cell proportions in the variation of gene expression in whole blood. At the same time, the model used by Decon-eQTL controls for the effects of the remaining cell types on gene expression. In this way, whole blood expression data, genotypes and (predicted) cell proportions can be integrated to assign a CTi effect from a bulk eQTL (Fig. 1).

**Fig. 3:**
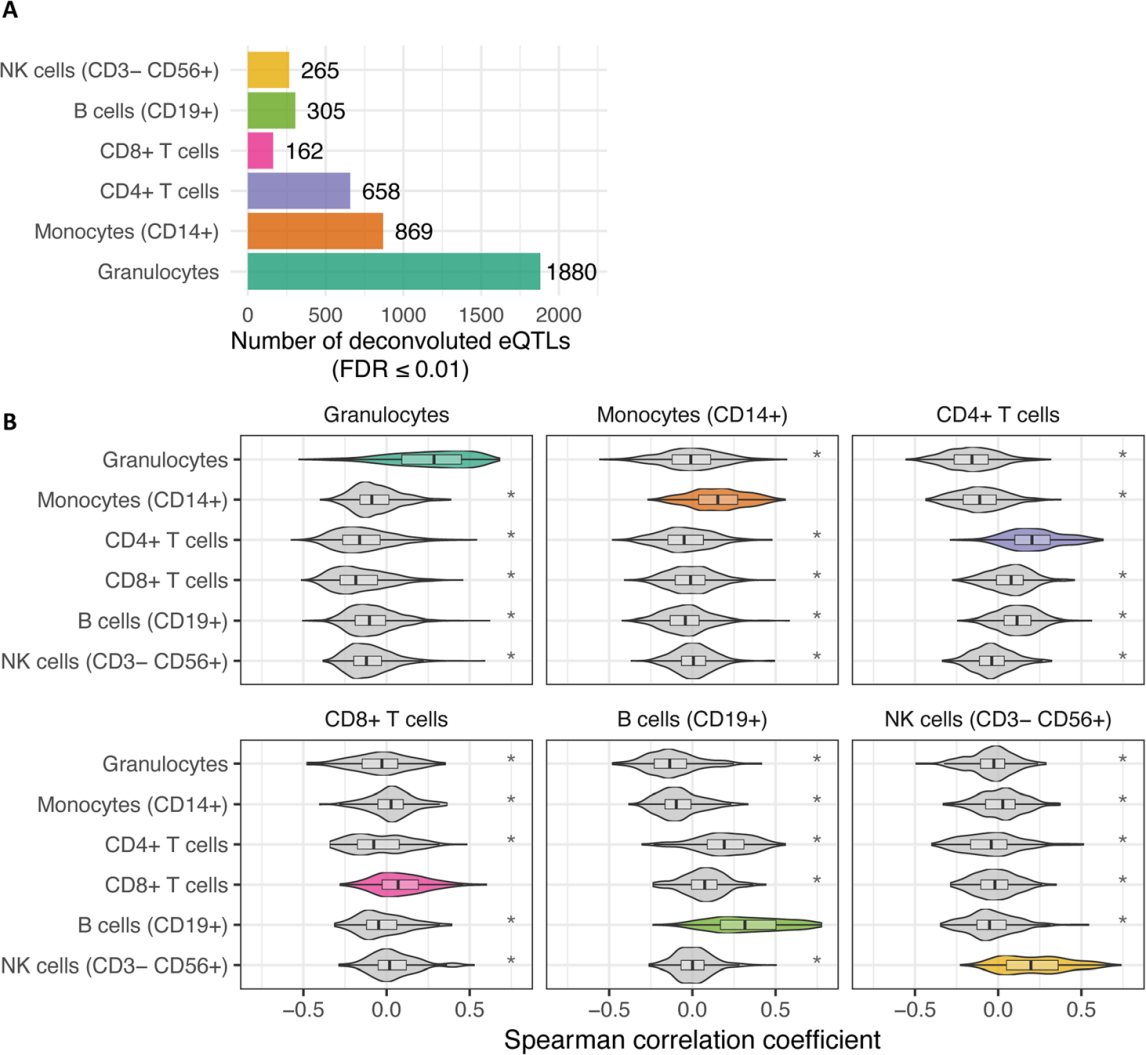
Deconvolution of whole blood eQTLs into CTi eQTLs. Decon-eQTL detects CTi eQTLs by integrating proportions of cell subpopulations (predicted by Decon-cell), gene expression and genotype information. **a** Number of deconvoluted CTi eQTLs in each cell type using whole blood RNA-seq data of 3189 samples in BIOS cohort. **b** Distribution of Spearman correlation coefficients between expression levels of CTi eQTL genes and cell counts for each cell subpopulation. The CTi eQTL genes show positive and statistically higher correlation (Spearman) with the relevant cell type proportions as compared to the rest (T-test *p*-value < 0.05) in an independent cohort (500FG)

We applied Decon-eQTL to 3198 samples (BIOS cohort) with transcriptome levels (RNA-seq), genotype information and cell proportions predicted by Decon-cell. Whole blood *cis*-eQTL mapping yielded 16,362 whole blood eQTLs (false discovery rate (FDR) ≤ 0.05). For each of these whole blood *cis*-eQTLs, we applied Decon-eQTL with a focus on 6 major cell subpopulations: granulocytes, CD14+ monocytes, CD4+ T-cells, CD8+ T-cells, B-cells and NK cells. These cell types were selected because the sum of their relative percentages was close to 100% and none of these cell type pairs had an absolute correlation coefficient R ≥ 0.75. Decon-eQTL computationally assigned 4139 CTi eQTLs from these subpopulations, reflecting 3812 genes and 3650 SNPs. 25% of the whole blood eQTLs have a significant (FDR ≤ 0.05) CTi eQTL effect given Decon-eQTL. The majority (31%) of the total CTi eQTL effects detected were found to be associated to granulocyte proportions, possibly because granulocytes comprise ~ 70% of circulating white blood cells (Fig. 3a). The majority (74%) of CTi eQTLs detected by our method were assigned to a single cell type (Supplementary Fig. 6A). Similarly, we find almost no sharing between cell types in single-cell eQTLs from 112 individuals. However, it should be noted that these eQTLs are likely not exclusively present for this particular cell type in biology, but that the statistical power given our sample size was sufficient to detect the interaction effects that we describe as CTi eQTL in this particular cell type. Decon-eQTL was only able to find a few cases of sharing of CTi eQTLs between cell types, likely due to a lack of power of the interaction model. An example of such a shared CTi eQTLs can be seen for the *NOD2 gene*, where Decon-eQTL detected a strong granulocyte-eQTL effect alongside a smaller opposite effect in CD14+ monocytes. This opposite effect has also been previously described in eQTL studies on purified CD14+ monocytes and neutrophils [8]. These results demonstrate that the effects of cell proportions on gene expression should be taken into account when interpreting eQTLs derived from bulk tissues.

### Decon-eQTL prioritizes genes to relevant cell types

CTi eQTL genes are expected to have higher expression levels in their relevant cell types, and their expression in whole blood should therefore be correlated with the proportions of these relevant cell types. To test this, we evaluated if the expression levels of the CTi eQTL genes detected in the BIOS cohort were correlated with their relevant cell proportions, and compared this to the correlation with non-relevant cell types. We calculated the Spearman correlation coefficients between the expression of the identified CTi eQTL genes and the measured cell proportions in the 500FG cohort (*n* = 89). We then compared the correlation coefficients we obtained here with those between expression and the remaining cell proportions. For each of the six cell subpopulations we evaluated in Decon-eQTL, their CTi eQTL genes had a significantly higher correlation with their relevant cell subpopulation than with other cell types (T-test, *p*-value < 0.05) (Fig. 3b). As such, this result shows a significant association between CTi eQTL genes and the proportion of their relevant CT in an independent cohort.

**Fig. 4:**
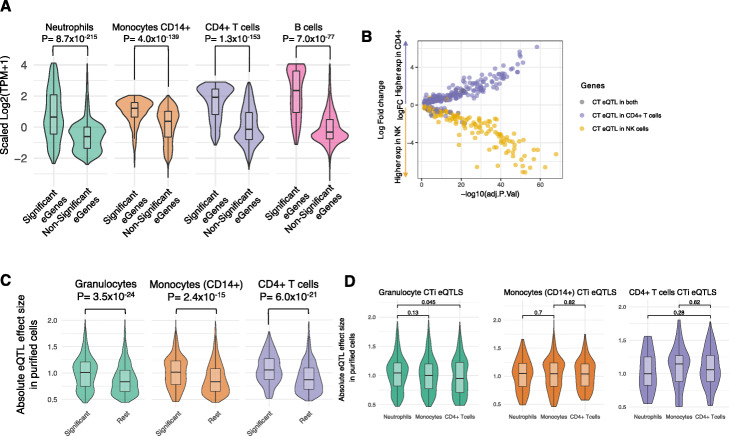
Validation of CTi eQTLs. **a** The expression of CTi eQTL genes in purified cell subpopulations from BLUEPRINT [23] are significantly higher in the relevant cell subpopulation when compared to other available cell subtypes (green for granulocyte eQTL genes showing expression for purified neutrophils; orange for monocytes; purple for CD4+ T cells; pink for B cells). **b** Genes differentially expressed (Adjusted p-value ≤0.5) between CD4+ T cells and NK cells are significantly enriched for CT eQTLs effects on CD4+ T cells (dots in purple, Fisher exact *P* = 1.8 × 10^17^) and NK Cells (dots in yellow, Fisher exact *P* = 2.3 × 10^18^), respectively. **c** CTi-eQTLs (FDR ≤ 0.05) show significantly larger effect sizes in the purified cell eQTL data [9] compared to the rest of the whole blood eQTLs for which we do not detect a cell type effect, as shown for deconvoluted granulocyte eQTLs in neutrophil-derived eQTLs (green),monocytes (orange) and CD4+ T cells (purple)

Next, we evaluated whether the significant CTi eQTL genes were over-expressed in their relevant cell subpopulation compared to eQTL genes that were found to be non-significant CTi eQTLs for the same cell type. For this purpose, we made use of the purified neutrophil, CD14+ monocyte, CD4+ T-cell and B-cell RNA-seq data from the BLUEPRINT dataset. We include these cell types because they were the only ones with more than three samples measured. For each of the four cell types, we observed that the expression of CT eQTL genes detected by Decon-eQTL was significantly higher (T-test, *p*-value ≤0.05) than the expression of non-significant Decon-eQTL genes (Fig. 4a). We also observed that the deconvoluted eQTL genes from granulocytes showed a relatively wider range of variation than the CT eQTL genes from the other three subpopulations. We hypothesized that this could be explained by the fact that granulocytes comprise ~ 70% of the cell composition in whole blood, thus giving us the power to detect eQTL for lowly expressed genes in granulocytes. This is partly supported by the observation that the variation of expression in whole blood of granulocyte CTi eQTL genes was significantly greater than for those CTi eQTL genes deconvoluted to the other five cell subpopulations (F-test, p-value ≤0.05, Supplementary Fig. 7).

Furthermore, by using publicly available transcriptome profiles (GSE78840 [27]) of purified NK cells and CD4+ T cells, we assessed if the differentially expressed genes across the two cell types were enriched for eGenes of deconvoluted CT eQTLs. Here we observed that the CD4+ differentially expressed genes (Adjusted *P*-value ≤0.05) were significantly enriched for CD4+ T cell eQTLs (Fisher exact *P* = 1.8 × 10^− 17^), whereas NK cell differential genes (Adjusted P-value ≤0.05) were significantly enriched for NK cell eQTLs (Fisher exact *P* = 2.3 × 10^− 18^) as shown in Fig. 4b.

In summary, we were able to show that the eQTL genes detected by Decon-eQTL are transcriptionally active in their relevant cell type because that is where they are more highly expressed.

### CT eQTLs identified by Decon-eQTL in whole blood are replicated in purified cell eQTL datasets

To validate the CT eQTLs defined by Decon-eQTL, we utilized eQTLs identified from purified neutrophils, CD4+ T-cells and CD14+ monocytes [9]. We first compared the absolute effect sizes of eQTLs from purified cells that are also significantly deconvoluted CTi eQTLs to the effect sizes of eQTLs from purified cells that are also non-significant deconvoluted CTi eQTLs for this cell type. For all three cell populations, effect sizes in our deconvoluted CTi eQTLs were significantly higher than the effect sizes of eQTLs without a significant CTi eQTL (Wilcoxon test, p-value ≤0.05, Fig. 4c). Next, we assessed the specificity of our deconvoluted CTi eQTLs by evaluating CTi eQTL effect sizes in non-relevant cell subpopulations. For example, we compared the effect sizes of deconvoluted granulocyte CTi eQTLs against those with non-significant deconvoluted granulocyte CTi eQTLs using the effect sizes of purified CD4+ T-cell eQTLs. Notably, we observed no statistically significant differences using effect sizes from non-relevant cell subpopulations (see off-diagonal comparisons in Supplementary Fig. 8), which further supports the biological relevance of our deconvoluted CTi eQTLs. However, when comparing the effect sizes in the purified eQTLs of only the CTi eQTLs that were significant across all three available cell subpopulations, we were not able to find significant differences (Fig. 4d). For example, the effect size of neutrophil CTi eQTLs is the same across neutrophils, monocytes CD14+ and CD4+ T cells*.*

To further demonstrate that the CTi eQTLs assigned by Decon-eQTL are biologically meaningful, we made use of the K27AC and K4ME1 epigenetic QTLs characterized in purified neutrophils, CD4+ T-cells and monocytes CD14+ [9]. In a similar fashion to the above comparison of effect sizes with purified eQTLs, we compared the absolute effect sizes from both K27AC and K4ME1 QTLs from eQTLs for which Decon-eQTL detects a significant CTi effect to the effect sizes of the other whole blood eQTLs. Here we observed that for corresponding cell types, e.g. evaluating granulocyte CT eQTLs in K27AC QTLs from purified neutrophils, the distribution of the absolute effect sizes is significantly higher for the chromatin mark QTLs (cmQTLs) than for non-significant CT eQTLs, which provides epigenetic evidence that our method is able to correctly assign cell type eQTL effects, as shown in the diagonal comparisons for both K27AC QTLS (Supplementary Fig. 9) and K4ME1 QTLs (Supplementary Fig. 10). Notably, for the non-relevant cell subpopulations, we observed that only one comparison (granulocytes vs. CD14+ monocytes) shows statistically significant higher effect sizes for K27AC QTLs and K4ME1 QTLs. For the rest of the non-relevant comparisons (shown in the off-diagonal of both Supplementary Fig. 9 and Supplementary Fig. 10), there are no statistically significant differences. Comparing the eQTL effect sizes in purified KC27AC and K4ME1 QTLs of only the significant CTi eQTLs across all three available cell subpopulations shows that the effect sizes from the relevant cell type are significantly stronger for all pairings except those between granulocytes and CD14+ monocytes (Supplementary Fig. 11).

**Fig. 5:**
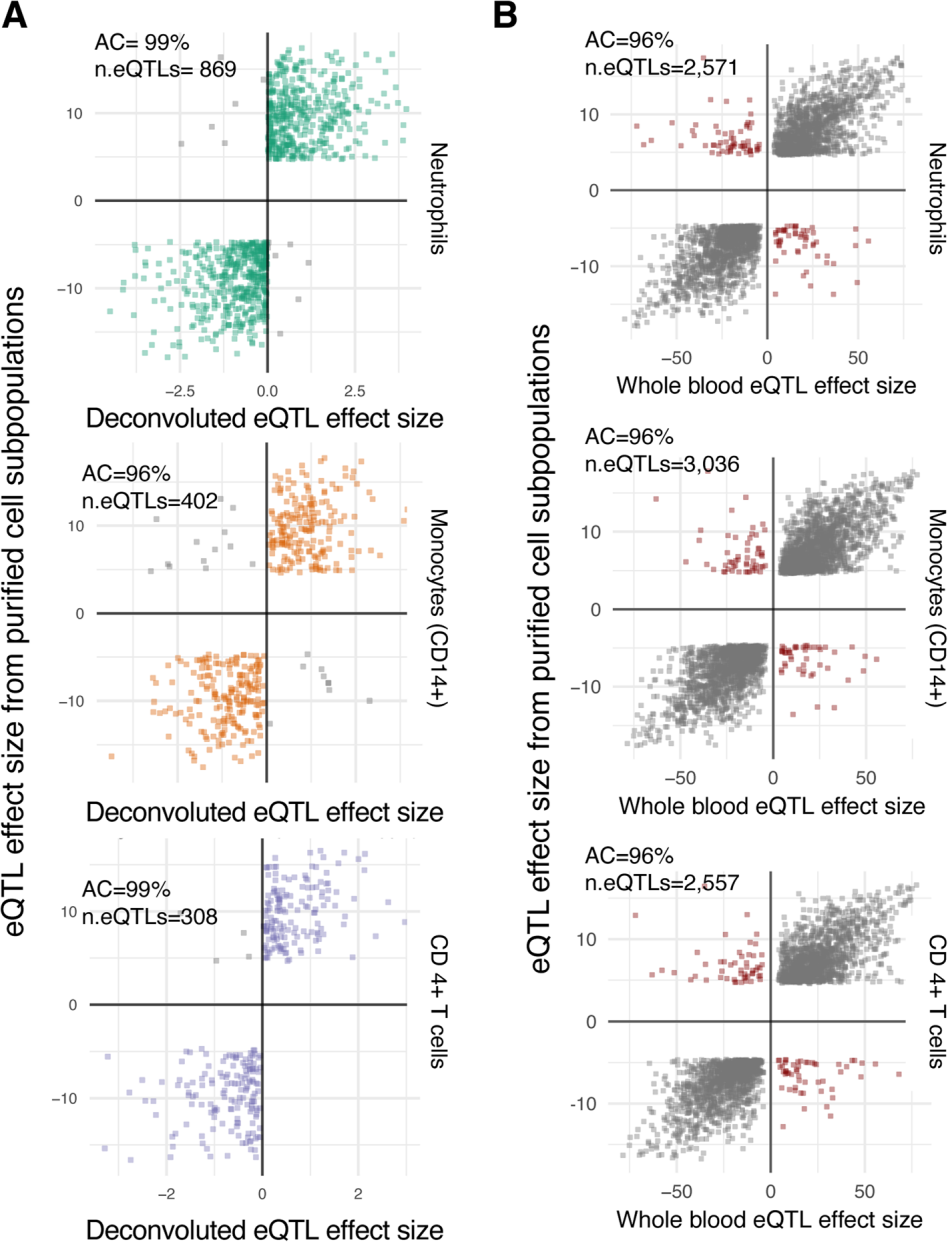
Allelic concordance of CTi eQTLs with eQTLs from purified cells. CTi eQTLs show high allelic concordance compared to eQTLs from purified cell subpopulations9. (**a**) for granulocyte eQTLs (green), CTi eQTLs achieved an allelic concordance of 99% compared to eQTLs from purified neutrophils. Similarly, the allelic concordances were 96 and 99% for CD14+ monocytes and CD4+ T cells, respectively. Except for monocytes, these values are higher than those observed for whole blood eQTLs when comparing to eQTLs from purified subpopulations, as shown in panel (**b**)

In addition to the comparison of effect sizes, we compared the allelic concordance between deconvoluted eQTLs and eQTLs from purified cell subtypes [9]. For each available cell type (neutrophils, CD14+ monocytes, and CD4+ T cells), we evaluated whether the direction of the eQTL effect on deconvoluted CT eQTLs was the same as the one observed from purified cell subpopulations. The allelic concordance between the deconvoluted eQTLs and purified eQTLs was high across cell types: 99% for granulocyte eQTLs (compared to neutrophil eQTLs), 96% for CD14+ monocytes eQTLs and 99% for CD4+ T cells (Fig. 5a). These rates of allelic concordance are significantly higher for granulocyte and CD4+ T-cell CTi eQTLs compared to those between whole blood eQTLs and eQTLs from purified cell subpopulations (Fig. 5b; Neutrophils, Fisher exact *p*-value = 3.91 × 10^6^; CD4+ T cells Fisher exact p-value = 0.005), whereas the allelic concordance for deconvoluted CD14+ monocyte eQTLs is the same as for whole blood eQTLs and purified CD14+ monocyte eQTLs (Fig. 5b). We also compared the allelic concordance of deconvoluted CTi eQTLs of specific cell types against the eQTLs of non-relevant purified subpopulations. Interestingly, the allelic concordance across non-relevant cell subtypes is consistently lower (off-diagonal Supplementary Fig. 12, Bonferroni-corrected Fisher exact p-value < 0.0001 for all comparisons). Higher allelic concordance across cell types was seen between deconvoluted granulocyte eQTLs and CD14+ monocyte eQTLs with a 95% allelic concordance, which shows that the direction of effect is often shared between related cell types.

Finally, we evaluated the allelic concordance rates for CTi eQTLs assigned by Decon-eQTL and K27AC QTLs from purified cell subpopulations. Here we observed a consistently high allelic concordance rate: 92% for granulocyte eQTLs (in purified Neutrophils), 87% for CD14+ monocytes and 92% for CD4+ T cells (boxed diagonal comparisons in Supplementary Fig. 13). These concordance rates are significantly higher than the ones between the whole blood eQTLs and K27AC QTLs from purified cell subpopulations (Supplementary Fig. 14) for neutrophils (Fisher exact test p-value = 9.06 × 10^− 14^), CD14+ monocytes (Fisher exact test p-value = 3.33 × 10^− 4^), CD4+ T cells (Fisher exact test p-value = 8.64 × 10^− 9^). Moreover, we noticed a consistent decrease in allelic concordance rates when assessing the concordance of CT eQTLs in K27AC QTLs of non-relevant cell subpopulations (off-diagonal comparisons, Supplementary Fig. 13). Taken together, the results from allelic concordance rates between deconvoluted CTi eQTLs and eQTLs/K27AC QTLs from purified cell subpopulations add a further layer of evidence to support the biological relevance of deconvoluted CT eQTLs.

### CTi eQTLs identified by Decon-eQTL in whole blood show high allelic concordance with single-cell RNA-seq eQTLs

**Fig. 6:**
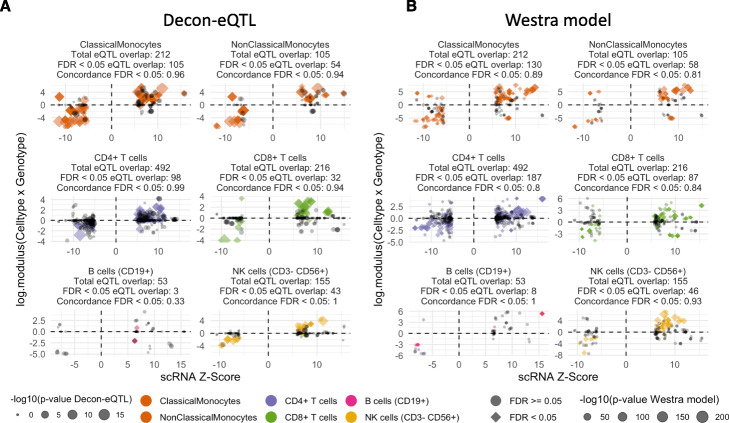
Allelic concordance of CTi eQTLs with eQTLs from single cell RNAseq. **a** Comparison in allelic direction between CTi eQTLs and eQTLs from single cell RNAseq experiments in 6 cell types. **b** Comparison in allelic direction between Westra model eQTLs and single cell eQTLs. In both panels coloured diamonds are FDR < 0.05, grey circles are FDR > = 0.0 in the single cell data, and the size is the -log10(p-value) of the predicted cell type interacting eQTLs

To replicate the deconvoluted CT eQTLs in the cell subtypes that were not available in Chen et al. [9] purified cell eQTLs, we utilized the recent single-cell RNA-seq eQTLs (sc-eQTLs) identified in CD14+ monocytes, NK cells, CD4+ T-cells, CD8+ T-cells and B-cells [24], as well as new single cell eQTL data that was processed in the same way. In total, we used sc-eQTLs from 112 individuals. We selected all significant eQTLs for each of the cell types (non-classical and classical monocytes were combined) and compared them to the direction of the eQTL effect given by Decon-eQTL, hereby observing an allelic concordance of 96.42% (Fig. 6a).

### Decon-QTL outperforms conventional interaction method

To our knowledge, our approach is the first to model the effect of multiple components of bulk blood RNA-seq simultaneously in an attempt to fully deconvolute gene expression levels into more precise cell type x genotype effects. Previous studies have used an interaction effect between genotype and cell proportions of one specific cell type to detect cell type eQTL effects using whole blood gene expression [10, 11], or used the correlation of the eQTL effect with cell type proxy genes [10, 11].

The Westra *et al* method has often been used to detect cell type eQTL effects using bulk expression data and cell proportions [28–31]. In brief, it focuses on the effect of the GxE interaction (where E represents cell proportions) to explain the variation in gene expression and only incorporates one cell type at a time. To properly compare Decon-eQTL with the ‘Westra method’, we applied both methods to the BIOS cohort and detected CT eQTLs for the six cell subpopulations. Replication of CT eQTLs identified by the Westra method was done as described above for Decon-eQTL. Here we observed that the eGenes (i.e. genes with eQTLs) detected by the Westra method show significantly higher expression for granulocytes (*p* = 3.0 × 10^− 12^, observed in purified neutrophils) and CD4+ T cells (*p* = 5.0 × 10^− 13^) and B cells (*p* = 5.1 × 10^− 11^), but not for CD14+ monocytes (*p* = 1, see Supplementary Fig. 15A). Next, we found that the distribution of effect sizes in eQTLs from purified cells is significantly higher for the CT eQTLs detected using the Westra method when compared to the rest of the whole blood eQTLs (*p* = 2.2 × 10^− 47^, *p* = 9.6 × 10^− 08^ and p = 1 × 10^− 47^ for neutrophils, CD14+ monocytes and CD4+ T cells, respectively; boxed-diagonal comparisons in Supplementary Fig. 15B), showing similar results to the ones from Decon-eQTL (Supplementary Fig. 8).

When we compared the allelic concordance rates between the direction of effects given by the interaction term from the Westra method to those found in eQTLs from purified cell subpopulations, we observed that the allelic concordances for granulocytes eQTLs (99%, evaluated in neutrophils, *p* > 0.05) and CD4+ T cells 93% (p > 0.05) (Supplementary Fig. 16) are comparable to those observed for Decon-eQTL (Fig. 4a). Conversely, the allelic concordance rate for CD14+ monocytes is only 62%, significantly lower than the results from Decon-eQTL (96%, *p* = 0.001). Finally, for granulocytes, CD4+ T cell eQTLs and monocytes, we overlapped the results from Westra method and Decon-eQTL with the eQTLs from purified cell types (Chen et al [9]) (Supplementary Fig. 17). For all three cell types, we found that Decon-eQTL is able to detect a larger number of eQTLs. For neutrophils, the Westra method has a higher replication rate (Fisher *p*-value = 0.002). For CD14+ monocytes, both methods had the same replication rate (Fisher p-value = 0.737). For CD4+ T-cells, Decon-eQTL had a better replication rate (p-value = 7.47 × 10^− 12^).

Finally, we compared the difference in allelic concordance with sc-eQTLs. The overall allelic concordance of Decon-eQTL CTi QTLs with sc-eQTLs (96.42%, Fig. 6a) is higher than that achieved by the Westra model (*p* = 1.235 × 10^− 08^), where we observed an overall allelic concordance of 84.67% (Fig. 6b). For both non-classical monocytes (Fisher p-value = 0.045) and CD4+ T-cells (Fisher p-value = 7.896 × 10^− 07^), Decon-eQTL has a significantly better allelic concordance. For CD8+ T-cells (Fisher p-value = 0.230), classical monocytes (Fisher p-value = 0.0513), B-cells (Fisher p-value = 0.055) and NK cells (Fisher p-value = 0.242), there is no significant difference. Nevertheless, Decon-eQTL shows a higher allelic concordance for NK cells, classical monocytes, and CD8+ T cells (93.8% vs 83.9, 96.2% vs 89.2, and 100% vs 93.5% respectively), while for B cells it has lower concordance (33% vs 100%).

Overall, these results demonstrate that Decon-eQTL is able to detect more CTi eQTLs that can be replicated in purified eQTL dataset than previously reported methods, especially in less abundant cell types such as CD14+ monocytes. However, the detection of interaction effects between genotype and cell proportions in order to dissect bulk (in this case whole blood) expression data and CTi eQTLs remains an area of great opportunity that could still be explored, particularly given the constantly increasing number of samples present in functional genomic cohorts and the growing numbers of purified and sc-eQTL datasets that can be used for validation.

## Discussion

We have developed a novel statistical framework, Decon2, that predicts the proportions of known immune cell subtypes using gene expression levels from whole blood (Decon-cell). These predicted cell proportions can then be used together with genotype information and expression data to deconvolute a whole-blood eQTL effect into cell type interacting effects (Decon-eQTL). Using a set of samples with both whole blood RNA-seq data and cell frequencies of 73 cell subpopulations, we demonstrated that Decon-cell was able to predict 34 independent cell subpopulations. The performance of Decon-cell has been validated in multiple independent cohorts and benchmarked with existing methods. The Decon-cell models were then applied to a cohort of 3189 samples for which whole blood RNA-seq data was available, resulting in predicted cell counts for these samples. By integrating bulk expression data, genotype and predicted cell counts of the BIOS cohort, Decon-eQTL was able to dissect whole blood eQTL effect into CTi eQTLs without purifying immune cell subpopulations. The results of Decon-eQTL were then validated again using several independent data types: 1) eQTLs from purified cell subpopulations, 2) chromatin QTLs of purified cells, 3) gene expression from purified cell types and 4) eQTLs derived from single-cell protocols. Compared with existing methods, Decon-eQTL consistently shows superior performance. To sum up, the proposed framework is useful for (re)-analyzing both existing and new bulk blood tissue datasets in order to detect CTi eQTL effects and can be applied and tested on other tissues once cell count proportions become available. Cataloging and further interpreting the role of CTi eQTLs will improve our understanding of the functional role of the SNPs associated with complex diseases at the level of specific cell subtypes.

The main advantage of our Decon-cell method for predicting cell proportions is that it does not rely on the gene expression measured in purified cell subtypes when defining signature gene sets. Moreover, our method does not require the definition of marker genes based on their differential expression compared to other cell subpopulations, unlike previously reported methods [12]. The signature genes defined by Decon-cell are determined using a completely unsupervised approach that applies regularized regression to select the optimal combination of genes to accurately predict a certain circulating cell proportion. The majority of these marker genes are differentially expressed across purified cell subpopulations, but not all. Nevertheless, these signature gene sets are still correlated to the cell proportions in whole blood. In summary, we have shown that Decon-cell can accurately predict the proportions of circulating immune cell subpopulations in three independent cohorts and that it out-performs previously reported methods within these cohorts.

Our Decon-eQTL method for detecting a CTi eQTL effect with bulk blood tissue expression data is, to our knowledge, the first attempt to simultaneously model whole blood gene expression profiles into its major components. In contrast to a previous method where single cell type (G x E) effects were evaluated one at a time [10, 31], Decon-eQTL incorporates all the major cell proportions simultaneously to better dissect the overall genetic effect of gene expression signal into cell subpopulation effects. We have shown that CTi eQTL genes found with Decon-eQTL have higher expression and higher effect sizes in purified neutrophils, CD14+ monocytes and CD4+ T-cells than do non-CTi genes, and we find significantly higher allelic concordance for two out of four tested cell types with sc-eQTLs than with a conventional interaction model (Fig. 6a and b). Moreover, we have also shown the biological relevance of the deconvoluted CTi eQTLs by validating our results on cmQTLs where CTi eQTLs have significantly higher effect sizes and allelic concordance rates are significantly higher than those of whole blood eQTLs. Finally, we have also demonstrated that Decon-eQTL can replicate sc-eQTLs derived from scRNA-seq data, showing a higher allelic concordance with sc-eQTLs than when using only whole-blood eQTL effects.

There are also limitations to our method. The CTi eQTLs detected by Decon-eQTL tend to be eQTL exclusive for the specific CT, suggesting that the CT with the strongest eQTL effect was selected by Decon-eQTL. This is likely due to the partial collinearity present between the CT proportions included in the model (as shown by their correlation structure in Supplementary Fig. 18A-B). Thus, the genetic effect of one cell type might be masked by another CT with a correlated cell proportion. The highest correlation coefficient among cell types included in the model was 0.75 (between granulocytes and B cells). Therefore, deconvoluting CTi eQTLs for partially correlated cell proportions could lead to false negative results for cell types with relatively weaker eQTL effects.

In our model, we included the six major blood cell types, but there are many more cell types available for which our method is not able to detect a CTi eQTL estimate. Furthermore, we only tested Decon-eQTL using genome-wide whole blood cis-eQTLs main effects. Such eQTL effects are very likely shared across multiple cell types, however we are only able to detect its interaction with only one cell type due to statistical power and co-linearity (Supplementary Fig. 6A), which is also seen in the sc-eQTLs with limited (112) samples (Supplementary Fig. 6B). Nevertheless, this does not imply that the CTi eQTL are exclusive for, or only present in, that specific cell type, as we observe in Fig. 4d, where the effect sizes of the significant CTi eQTLs in purified subpopulations are not significantly different across all three purified cell subpopulations. Yet this difference in the effect-size of CTi eQTLs between relevant and non-relevant cell types can be seen in histone modification QTLs (as shown in Supplementary Fig. 11), likely due to the cell type-specificity of epigenetic marks. Lastly, Decon2 has only been tested in whole blood, where large numbers of samples are available, and therefore it is not known how it will perform in other tissues.

The proposed framework of Decon2 is generic for predicting cell subpopulations in bulk tissues (Decon-cell) and re-distributing the overall eQTL effect into cell types (Decon-eQTL). Both methods have been implemented in freely available software. In both the R package and the user interface-based webtool, we provide the models for predicting cell subpopulation in whole blood that were constructed and validated in this work so that interested users can estimate immune cell subpopulations in whole blood in healthy people of western European ethnicity, as our models were built using a Dutch cohort (500FG).

## Conclusion

In summary, Decon2 is a computational method that can accurately assign CT effects in whole blood functional genomic cohorts. It can be applied to any dataset for which genotypes and expression data is available and could potentially aid in understanding the molecular effects of genetic risk factors associated with complex diseases at the cell-subpopulation level. Our method makes it possible to create CT gene regulatory networks that could explain the different effects that each CT has on a complex disease in a cost-efficient way. Since Decon2 only requires gene expression and genotype information to deconvolute bulk blood eQTLs into CTi eQTLs, it is possible to re-analyze existing bulk blood RNA-seq data for which genotypes are also available. In this scenario, we would use Decon-cell to predict cell proportions in whole blood and obtain CT information on many more eQTLs through an increase in sample size. In addition to whole blood, the methods behind Decon2 can potentially be generalized to use transcriptional profiles derived from any other type of bulk tissue, such as biopsies from tumors or other solid tissues implicated in complex disease etiology. However, the method has not yet been tested in other tissues. Our methods can hence aid in the detection of genetic effects on gene expression in rare cell subpopulations in bulk tissues.

## Methods

### RNA-seq data collection in 500FG cohort

We selected a representative subset of 89 samples from the 500 participants of the 500FG cohort, which is part of the Human Functional Genomics Project (HFGP). Our subset was balanced for age and sex based on the original distribution in the cohort. RNA was isolated from whole blood and globin transcripts were subsequently filtered by applying the Ambion GLOBINclear kit. The samples were then processed for sequencing using the Illumina TruSeq 2.0 library preparation kit. Paired-end sequencing of 2 × 50-bp reads was performed on the Illumina HiSeq 2000 platform. The quality of the raw reads was checked using FastQC (http://www.bioinformatics.babraham.ac.uk/projects/fastqc/). Read alignment was performed with STAR 2.3.0 [32, 33] using the human Ensembl GRCh37.75 as reference, and the aligned reads were sorted using SAMTools [34]. Lastly, gene-level quantification of the reads was done using HTSeq [35].

### RNA-seq preparation and data processing in the BIOS cohort

RNA was isolated from whole blood and globin transcripts were subsequently filtered by applying the Ambion GLOBINclear kit. Library preparation was performed using the Illumina TruSeq v2 library preparation kit. Next, Illumina HiSeq 2000 was used for paired-end sequencing of 2 × 50 bp reads while pooling 10 samples per lane and expecting > 15 million read pairs per sample. Read sets were generated using CASAVA, retaining only those reads that passed Illumina Chastity Filter.

Quality control of the reads was evaluated using FastQC (http://www.bioinformatics.babraham.ac.uk/projects/fastqc/). Adaptor sequences were trimmed out using cutadapt (v1.1) with default settings. Low quality ends of reads were removed using Sickle (v1.200) (https://github.com/najoshi/sickle).

Reads were then aligned using STAR 2.3.0e [33]. All SNPs present in the Genome of the Netherlands (GoNL) with MAF ≥ 0.01 were masked from the reads to avoid reference mapping bias. Read pairs with at most eight mismatches and mapping to at most five positions were used. Quantification of counts per genes was done using Ensembl v.71 annotation (which corresponds to GENCODE v.16).

### Genotype data of the BIOS cohort

Genotype information was independently generated for each of the cohorts, further details on data collection and methods used for genotyping can be found in their papers (CODAM [36], LLDeep [16], LLS [17], RS [18] and NTR [21]).

Genotypes were harmonized to GoNL with Genotype Harmonizer [37] and imputed with IMPUTE2 [38] using GoNL as reference panel. SNPs with an imputation score below 0.5, a Hardy-Weinberg equilibrium *P*-value smaller than 1 × 10^− 4^, a call rate below 95%, or a MAF smaller than 0.05 were filtered out. For further analysis, only eSNPs from whole blood *cis*-eQTL top effects were subsequently used in Decon-eQTL.

### Quantification of cell proportions in 500FG cohort

Inclusion criteria and further description of the participants of the 500FG cohort can be found at http://www.humanfunctionalgenomics.org. A total of 73 manually annotated immune cell subpopulations were quantified using 10-color flow cytometry. To minimize biological variability, cells were processed immediately after blood sampling and typically analyzed within 2–3 h. Cell populations were gated manually as previously described [14].

### Cis-eQTLs in the BIOS cohort

For *cis*-QTL mapping, we tested for association between genes and SNPs located within 250 kb of a gene center. SNPs with MAF ≥ 0.01, call rate = 1 and Hardy-Weinberg equilibrium *p*-value ≥0.0001 were included. eQTLs were declared to be significant at FDR < 0.05. Pre-processing of RNA-seq and QTL mapping was performed using a custom eQTL pipeline that has been described previously [11].

### Normalization and correction of gene expression data for deconvolution of eQTL effects

Total read counts from HTSeq were first normalized using the trimmed means of M (TMM) values32. TMM expression values were then log2 transformed. For predicting cell proportions, we used scaled expression data in both the 500FG and BIOS cohorts.

For the deconvolution of eQTLs, the expression was log2 transformed and corrected for the effects of cohort, age, sex, GC content, RNA degradation rates, library size and number of detected genes per sample using a linear model. The corrected expression data was then exponentiated to maintain the original linear relationship across read counts (gene expression) and cell proportions.

### General description of Decon2

Decon2 is a statistical framework for estimating cell counts using molecular profiling such as expression data from heterogeneous samples (Decon-cell) and consecutive deconvolution of expression quantitative trait loci (Decon-eQTL) into each cell subpopulation. To predict cell proportion levels using Decon-cell built in models, it’s only input is a matrix As input Decon-cell takes a table of normalized gene expression counts, with samples as columns and genes as rows, and outputs a table of predicted cell count proportions for cell types that were included in the training model.. Decon-cell also enables the user to generate its own custom models, for which it requires a matrix of gene expression to train the model and a matrix of measured cell proportions; this will output a list with one specific model for each of the cell types included. A matrixtable of normalized gene expression levelscounts, a matrixtable of predicted or measured cell count proportions, and a matrixtable of genotype dosages (0 for homozygous reference, 1 for heterozygous, and 2 of homozygous alternative), lastlyand a table with the SNP + gene combinations to test, are used as input for Decon-eQTL, and this outputs for each SNP + gene combination the beta and p-value of the cell-type dependent eQTL effect. See supplemental Fig. 20 for a graphical overview.

### Prediction of cell proportions using gene expression levels from bulk tissue (Decon-cell)

For cell count prediction, expression data is TMM normalized, log2(expression+ 1) transformed and z-transformed (scaled). We proposed that the abundance of molecular markers such as gene expression could be used as proxies to predict cell proportions. This can be represented as:

1 $$ {C}_{kj}={\beta}_{ki}\ {Y}_{ij}+{e}_{kj} $$

where expression data is Y_*ij*_ for genes *i* = 1, 2, …, G and samples *j* = 1, 2, …, *N* and cell count data is C_*kj*_ for sample *j* in cell type *k* (k = 1, 2, …, K). β_*ki*_ represents the coefficients of gene *i* in determining cell counts of cell type *k* of a complex tissue. e_*kj*_ is the error term.

In order to select only the most informative genes for predicting cell counts, we implemented a feature selection scheme by applying an elastic net (EN) regularized regression [26]. In the EN algorithm, the *β*_*k*_ *Y* are estimated by minimizing:

2 $$ {\left\Vert\ {C}_k-{\beta}_kY\ \right\Vert}^2\  subject\ to\ \left(1-\alpha \right)\ {\left\Vert\ {\beta}_k\ \right\Vert}^2+\alpha {\left\Vert {\beta}_k\right\Vert}_1\le s $$

*s* is a tuning parameter that limits the number of features that will be included in the final predictor model. We estimate the best *s* per cell type by applying a 10-fold cross-validation approach, where the most optimal penalty parameter (*α*) was obtained.

### Deconvolution of eQTL effects (Decon-eQTL)

Decon-eQTL models the expression level in the bulk tissue by considering the genetic contribution of multiple cell types present in the system. For identifying the CT eQTL effect, the interaction term between a particular cell type and genotype was tested for statistically significant contribution to the explained variance on the expression levels of particular genes, while accounting for the remaining cell proportions. If we consider a generic eQTL linear model for whole blood it can be described as:

3 $$ y=a+\beta .g+e $$

where *y* is the measured gene expression, *a* the modeled non-genetic dependent expression, *g* the genotype coded as 0, 1 or 2, *β*. *g* the genotype-dependent expression and *e* the error, e.g. unknown environmental effects. Here, all three terms are modeling the effect of the mixture of different cell types present in blood. In an RNA-seq-based gene expression quantification of a bulk tissue, one could express gene expression levels (*y*) as the sum of counts (*ψ*) per *K* cell types:

4 $$ y={\sum}_{k=1}^K{\psi}_k $$

For every cell type, the expression level can be written as a generic eQTL model (eq. 3) weighted by the cell proportions. *ψ*_*k*_ is a combination of the genetic and non-genetic contribution of the cell type to *y*. The non-genetic contribution per cell type is *β*. c, where c is the cell count proportions. The genetic contribution is *β*_*k*_. *g* : *c*_*k*_. For *k* cell types the expression is then:

5 $$ y={\sum}_{k=1}^K{\psi}_k={\varSigma}_k.\left(\ {\beta}_k.{c}_k\right)+{\varSigma}_k.\left({\gamma}_{k.}\ g\times {c}_k\right)+e $$

where *y* is the measured expression levels, *k* is the total number of cell types, *c*_*k*_ is the cell count proportions of cell type *k*, *g* is the genotype and *e* is the error term. Since we are assuming a linear relationship between total gene expression and the levels of expression generated by each of the cell types composing a bulk tissue, the cell proportions are scaled to sum to 100% such that the sum of the effect of the cell types equals the effect in whole blood. Here we assume that the true sum of the cell counts should be very close to 100% of the total PBMC count, which is why we include the 6 cell types that together form the top hierarchy given the gating strategy used to quantify the cell subpopulations [14]. The genotype main effect is not included in the model because the sum of the genotype effect per cell type should approximate the main effect.

Because the contribution of each of the cell types to expression level *y* cannot be negative, we constrain the terms of the model to be positive using Non-Negative Least Squares [39, 40] to fit the parameters to the measured expression levels. However, if the allele that has a negative effect on gene expression is coded as 2, the best fit would have a negative interaction term, which would be set to 0. To address this, we want the allele that causes a positive effect on gene expression to always be coded as 2. However, the effect of an allele can be different per cell type, therefore the coding of the SNP should also be different per cell type. We therefore run the model multiple times, swapping the genotype encoding for one of the interaction terms each time. The encoding that gives the lowest R-squared is then chosen as the optimal genotype encoding. For the encoding, we limit the number of genotypes that have an opposite genotypic encoding to a maximum of one interaction term, as we have observed that this leads to no significant difference when compared to using all possible configurations and limits the number of models that have to be run from k^2^ to (2*k) + 2.

To test if there is a CT interaction effect, we run the linear model of eq. 5 and, for each CT, run the same model with the cell proportion:genotype interaction term removed. For example, when testing two cell types the full model is:

6 $$ y={\beta}_1.{c}_1+{\beta}_2.{c}_2+{\gamma}_1.g\times {c}_1+{\gamma}_2.g\times {c}_2+e $$

and the two models with the interaction terms removed are:

$$ y={\beta}_1.{c}_1+{\beta}_2.{c}_2+{\gamma}_1.g\times {c}_1+e $$

7 $$ y={\beta}_1.{c}_1+{\beta}_2.{c}_2+{\gamma}_2.g\times {c}_2+e $$

For both the full model and the CT models, we calculated the sum of squares using the different genotype configurations detailed above. For both the full and the CT models, we then selected the genotype configuration with lowest sum of squares. Then, for each CT, we tested if the full model could significantly explain more variance than the CT model using an ANOVA.

We then applied our strategy to 16,362 significant whole blood cis-eQTL top effects detected using the BIOS cohort. We then correct the *p*-values for multiple testing using FDR for each of the cell types, i.e. granulocyte eQTL p-values were corrected for 16,362 tests in the same way as CD4+ T cells eQTL p-values were corrected for the exact same number of tests.

### Westra et al. interaction model

In the Westra et al. model, expression data is normalized in the same way as in Decon-eQTL. The effect of the cell type is predicted using a genotype * cell count interaction term:

$$ y=I+{\beta}_1.G+{\beta}_2.c+{\beta}_3.\mathrm{c}\ x\ G+e $$

where y is expression, *I* the intercept, G the genotype, c the cell count and c x G the cell count x genotype interaction term. Additional restrictions are set on the p-values. For neutrophils, if (the *β* of the neutrophil *x G* interaction term) * (the *β* of the *G* interaction term) < 0, the p-value is set to 1. For CD4+ and monocytes, if (the *β* of the neutrophil *x G* interaction term) * (the *β* of the *G* interaction term) > 0, the p-value is set to 1.

### Comparison between allelic concordance

For the comparison between allelic concordances, we counted the concordant and discordant eQTLs for each of the cell type comparisons and did a Fisher exact test between each of the groups. The p-values are Bonferroni-corrected.

### Single-cell eQTLs

The sc-eQTLs were obtained for 112 individuals in the same way as described in Van der Wijst et al. [24] For the allelic direction comparison, we used all significant eQTLs. Classical monocyte and non-classical monocyte eQTLs were combined and jointly compared to Decon-eQTL Monocytes.

## Supplementary information

**Additional file 1 : Supplementary Figure 1:** Prediction performance of Decon-cell within 500FG: The Y-axis represents the 73 immune cell types quantified by FACS in the 500FG cohort. The bar plot on the left panel shows the mean Prediction Performance (Spearman correlation coefficient between predicted and measured cells across 100-fold cross validations). On the right panel, box plots represent the distribution of the Prediction Performance within 100 iterations of the cross validations. A cutoff of mean Prediction Performance ≥0.5 was applied to define predictable cell types (green). **Supplementary Figure 2.** Signature genes selected for prediction of cell proportions by Decon-cell: **(A)** Total number of marker genes (genes selected in ≥80% of all models in the 100 iterations) per predictable cell type. Different colors indicate different subpopulations. **(B)** The number of genes significantly correlated with cell counts (Spearman correlation, adjusted *P* ≤ 0.05) (y-axis) shows the total number of significantly correlated genes, while the x-axis shows the prediction performance (x-axis). **(C)** Distributions of the total number of “strongly” correlated genes (absolute Spearman correlation ≥0.3) between predictable and unpredictable cell subpopulations. **Supplementary Figure 3.** Comparison of prediction performance between Decon-cell and other existing methods. (A) Performance of Decon-cell: the measured (x axis) and predicted cell proportions (y-axis) were compared for neutrophils (given by granulocytes in 500FG), lymphocytes and monocytes CD14+ and granulocytes in three independent cohorts (shown by row, from top to bottom: LLDeep (*n* = 627); LLS (*n* = 660); RS (*n* = 773)). (B) Comparison of prediction performance for Decon-cell, CIBERSORT and xCell in three independent cohorts for a total of 4 major immune subpopulations. **Supplementary Figure 4**. Prediction performance of xCell and CIBERSORT in three independent Dutch populations (LLDeep, n = 627; LLS, n = 660; RS, n = 773)**.** (A) Scatter plots showing the measured cell proportions of circulating immune cells on the x-axis and the xCell enrichment score on the y-axis. (B) Scatter plots showing the measured cell proportions of circulating immune cells on the x-axis and the predicted cell proportions given by CIBERSORT) on the y-axis. **Supplementary Figure 5.** Expression of marker genes selected by Decon-cell. Expression levels (scaled, log2(TPM + 1)) of signature genes in the data in three purified cell subpopulations: CD4+ T cells **(A)**, neutrophils/granulocytes **(B)** and monocytes **(C)** in the data from BLUEPRINT. Cell subpopulations are indicated in different colors by columns. Correlation of each of the signature genes and the cell subpopulation percentage in the 500FG cohort is shown on by the green bar at the left-hand side of heatmap figure, i.e. darker green corresponds to higher correlations. **Supplementary Figure 6.** Many of the CTi eQTL are cell type exclusive. Colored bar plot on the left shows the total number of significant CTi eQTLs in whole blood eQTLs (as also shown in Fig. 2a). Gray bar plot shows the total number of eQTLs shared across the possible combinations of the six cell subpopulations under study. **Supplementary Figure 7.** Variation of gene expression across samples for deconvoluted cell-type eQTLs genes in whole blood. Granulocyte eQTL genes show significantly higher variance across the BIOS samples (F test *p*-value ≤0.05) compared to those from monocytes, CD4+ T cells, CD8+ T cells, B cells and NK cells. **Supplementary Figure 8**. Validation of CTi eQTLs using effect sizes of eQTLs from purified cells. CTi eQTLs (FDR ≤ 0.05) from the BIOS cohort show a significantly bigger effect size in purified cell eQTLs [9] from their relevant cell subtype as compared to other whole blood eQTLs (diagonal boxed comparisons). The off-diagonal comparisons show that these eQTL genes are specific to a cell subpopulation because the differences in effect sizes are non-significant in all but one case (CD4+ T cell eQTL genes in monocyte-derived eQTLs). **Supplementary Figure 9**. Validation of CTi eQTLs using effect sizes of K27AC QTLs from purified cells. CTi eQTLs (FDR ≤ 0.05) show a significantly bigger effect size for K27AC QTLs that have peaks located in the promoter region of the eGenes from their relevant cell subtype compared to the rest of the significant whole blood eQTLs (diagonal boxed comparisons). The off-diagonal comparisons show that these eQTL genes are specific to a cell subtype because the differences in effect sizes are non-significant in all but the comparisons across Neutrophils and Monocytes (CD14+). **Supplementary Figure 10**. Validation of CTi eQTLs using effect sizes of K4ME1 QTLs from purified cells. CTi eQTLs (FDR ≤ 0.05) show a significantly bigger effect size for K4ME1 QTLs (where the eGenes is the closest gene tagging the K4ME1 QTLs peak) from their relevant cell subtype compared to the rest of the significant whole blood eQTLs (diagonal boxed comparisons). The off-diagonal comparisons show that these eQTL genes are specific to a cell subtype because the differences in effect sizes are non-significant in all but the comparisons between neutrophils and monocytes (CD14+). **Supplementary Figure 11**. Validation of CTi eQTLs using allelic concordance with eQTLs results from purified cells. CTi eQTLs (FDR ≤ 0.05) show high allelic concordance with their respective purified cell eQTLs. Top row shows allelic concordance of deconvoluted granulocyte eQTLs (all in green) against neutrophils, monocytes and CD4+ T cells. Second row shows deconvoluted monocyte eQTLs against purified cell eQTLs in the same order as the top row. Bottom row shows the same comparisons as for deconvoluted CD4+ eQTLs. Allelic concordance of the off-diagonal (comparing CTi eQLTs with non-relevant cell types) show a consistent decrease in allelic concordance. *P*-values are Bonferroni-corrected Fisher exact tests between groups. **Supplementary Figure 12**. Validation of CTi eQTLs using allelic concordance with K27AC results from purified cells. CTi eQTLs (FDR ≤ 0.05) show a high allelic concordance in their respective purified cell K27AC QTLs. Top row shows allelic concordance of deconvoluted granulocyte eQTLs (all in green) against neutrophils, monocytes and CD4+ T cells derived from K27AC QTLs. Second row shows deconvoluted monocyte eQTLs (all in orange) against purified cell K27AC QTLs in the same order as top row. Bottom row shows the same comparisons as for deconvoluted CD4+ eQTLs (all in purple). Allelic concordance of the off-diagonal (comparing deconvoluted eQTLs with non-relevant cell types) show a consistent decrease in allelic concordance when compared to the relevant cell type comparisons. P-values are Bonferroni-corrected Fisher exact tests between groups. **Supplementary Figure 13**. Allelic concordance between whole blood eQTLs and K27AC QTLs for purified neutrophils, CD14+ monocytes and CD4+ T cells. **Supplementary Figure 14.** Comparison of whole blood eQTLs with eQTLs from single cell RNA-seq Whole blood eQTLs show 89% allelic concordance for significant eQTLs derived from scRNA-seq data, comprising monocytes CD14+, B cells, CD4+ T cells, CD8+ T cells and NK cells. **Supplementary Figure** 15 Validation of cell type eQTLs detected in the BIOS cohort using the Westra et al method: (A) Expression of eGenes in purified cell subpopulations from BLUEPRINT (green for granulocyte eQTL genes showing expression for purified neutrophils; orange for monocytes; purple for CD4+ T cells; pink for B cells). (B) CT eQTLs detected by the Westra method show a significantly larger effect size in purified cell eQTLs [11] as compared to the rest of the whole blood eQTLs. Boxed-diagonal shows the comparisons with relevant cell types where the effect differences are stronger. **Supplementary Figure** 16 Allelic concordance rates of cell type eQTLs detected using the Westra et al method and eQTLs from purified cells. Top row shows allelic concordance of granulocyte CT eQTLs against neutrophils, monocytes and CD4+ T cells. Second row shows CT monocyte eQTLs against purified cell eQTLs in the same order as top row. Bottom row shows the same comparisons for CT CD4+ eQTLs. **Supplementary Figure** 17 Comparison of Decon-eQTL with Westra et al method. Overlap of CT eQTLs detected with Decon-eQTL and the Westra et al method and those found to be significant in purified cell subpopulations for granulocyte QTLs (A), CD4+ T cells (B), and monocytes (C). **Supplementary Figure 18** Distribution and correlation among circulating cell proportions. (A) Scatter plots show the correlations between different cell subpopulations in 89 samples from 500FG. Blue line indicates a fitted linear model. Diagonal plots depict the overall density distribution per cell type. Upper right triangle shows the Pearson correlation coefficient for each pairwise comparison. (B) Correlations between different cell subpopulations in the BIOS cohort obtained by prediction using Decon-cell. **Supplementary Figure 19.**General overview of the Decon2 method. (A) Gene expression can be used to predict cell count percentages of cell counts that are already trained in the Decon-Cell model. Additionally, the model can be trained on different cell types if expression data and cell count proportions are available. (B) Decon-eQTL models the cell type dependent eQTL effect using expression, genotype, and measured cell count proportions or, if unavailable, predicted cell count proportions.

**Additional file 2 : Supplementary Table 1:** Ensembl IDs and symbol names of the marker genes selected by Decon-cell for the 34 predictable circulating immune cell proportions.

**Additional file 3 : Supplementary Table 2:** Summary statistics from Decon-eQTLs for the 16,362 whole blood eQTLs.

## Data Availability

The deconvolution summary statistics are made available as supplementary table. Information on how to request the genotype and RNAseq data used for the eQTL calculation can be found here: https://www.bbmri.nl/acquisition-use-analyze/bios. A subset of the single cell eQTLs is preliminary data for which a manuscript is in preparation, and will be made available after publication of that manuscript. Contact Lude Franke (l.h.franke@umcg.nl) to request access to this data. The GEO accession code for the expression data of 500FG is GSE134080.

## Abbreviations

eQTL: expression quantitative trait loci
CT: Cell type
CTi: Cell type interaction
GWAS: Genome-wide association studies
sc: single cell
GxE: Gene by environment interaction
TMM: Trimmed means of M
