# Supplementary material for: Deconvolution of bulk blood eQTL effects into immune cell subpopulations
Source: BMC Bioinformatics. 2020 Jun 12;21:243. doi: 10.1186/s12859-020-03576-5 (PMC7291428; doi:10.1186/s12859-020-03576-5)

Supp. Figure 1.

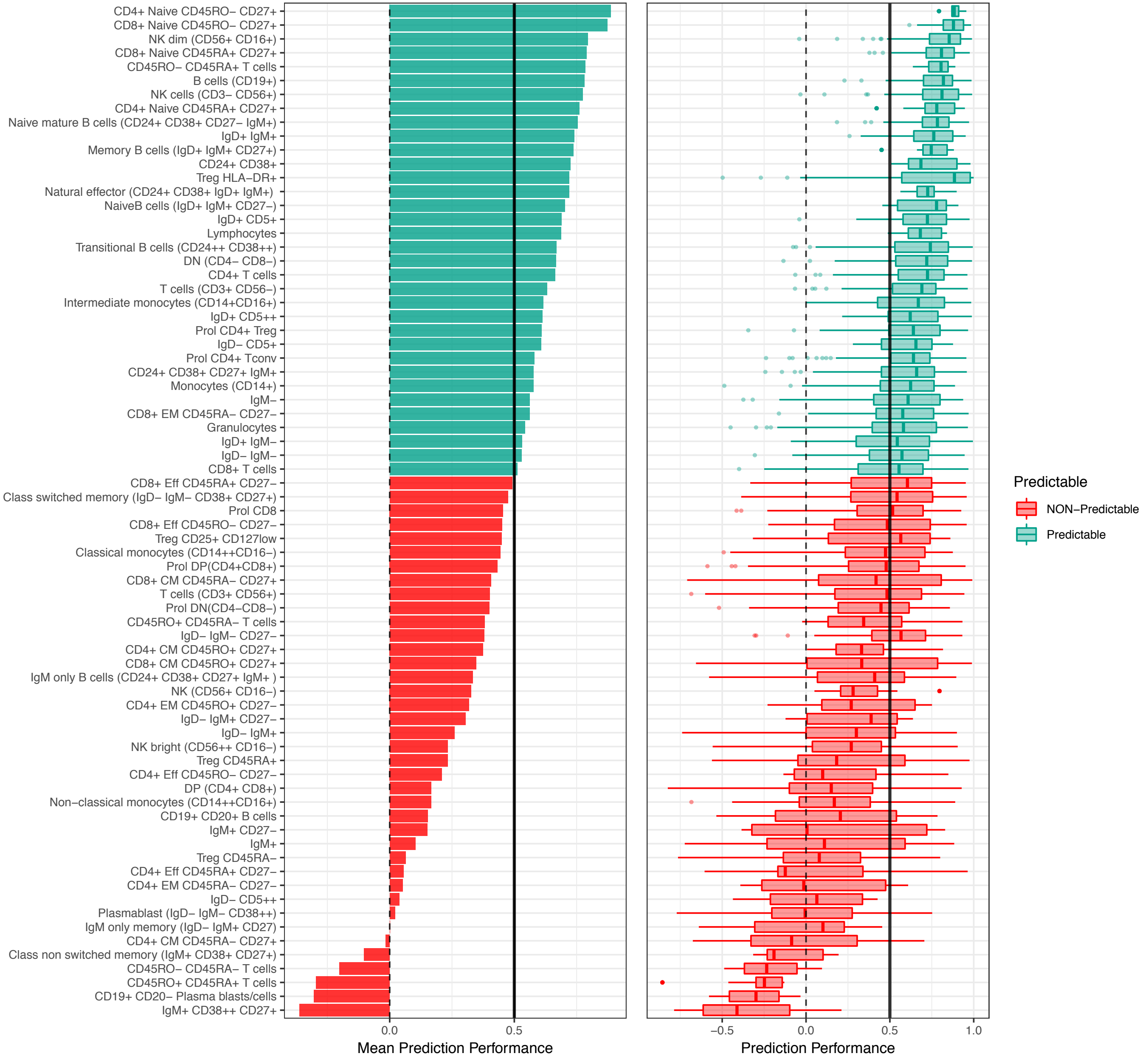

Supp. Figure 2.

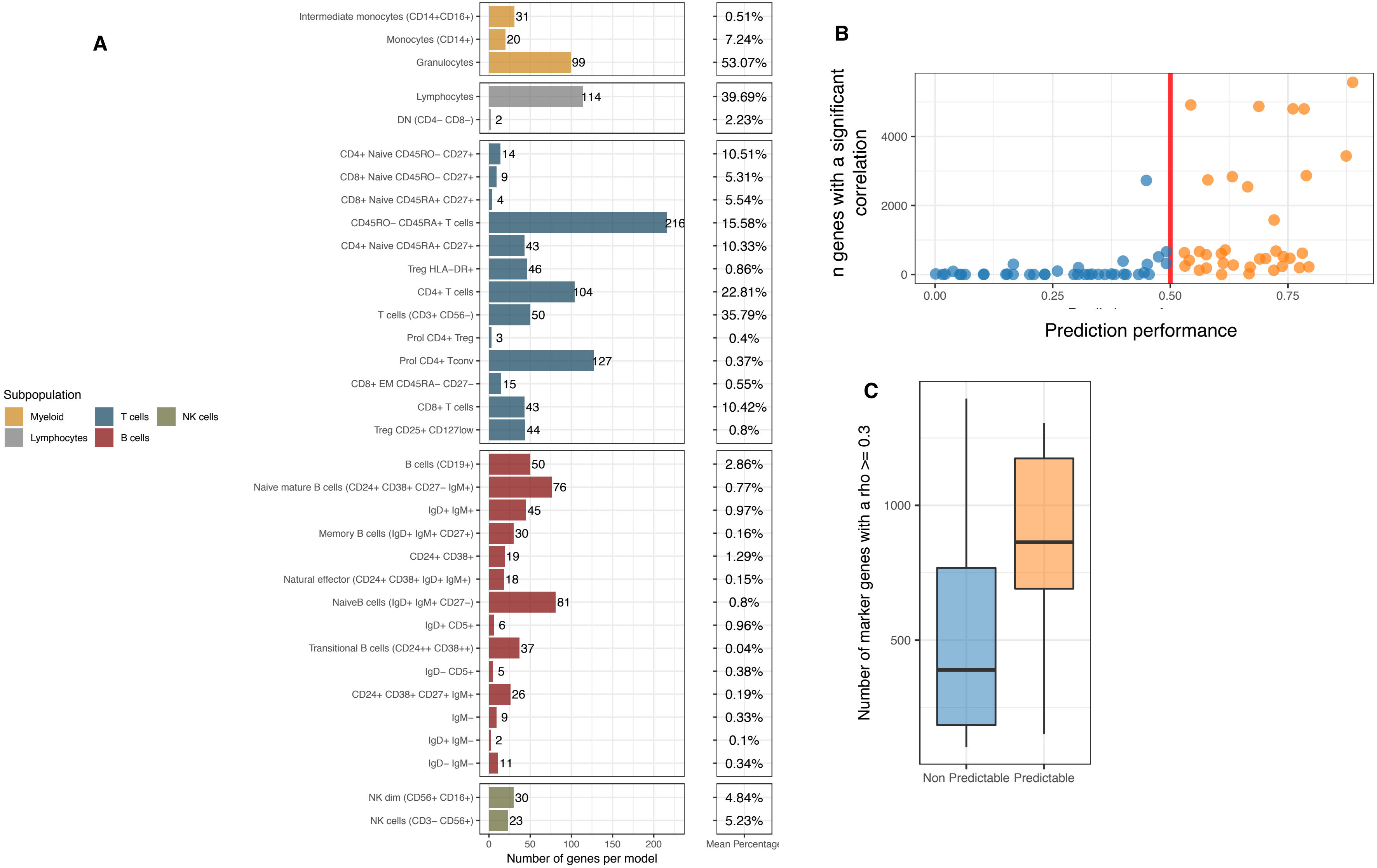

Supp. Figure 3.

A

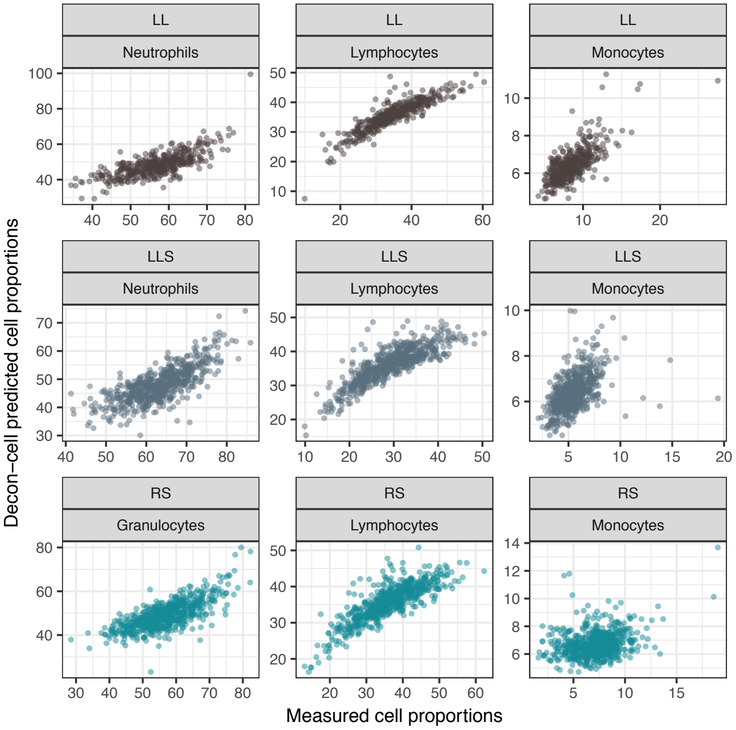

B

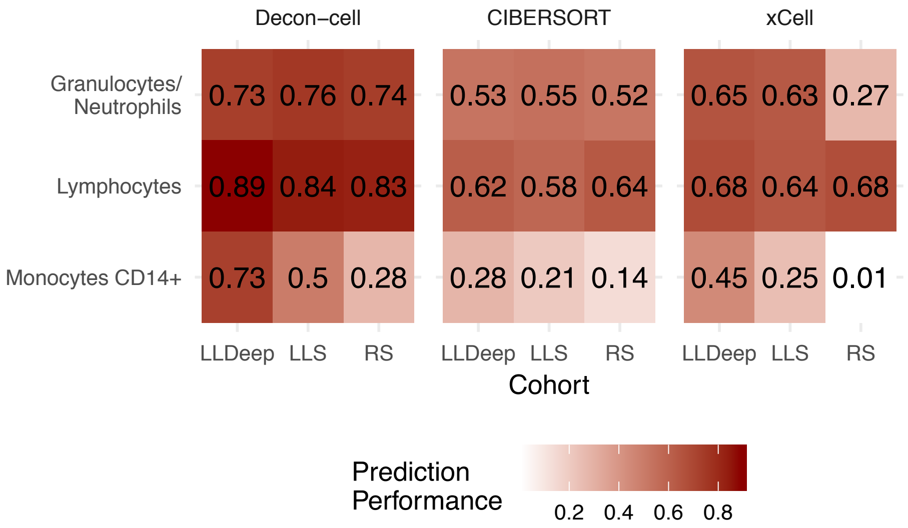

Supp. Figure 4.

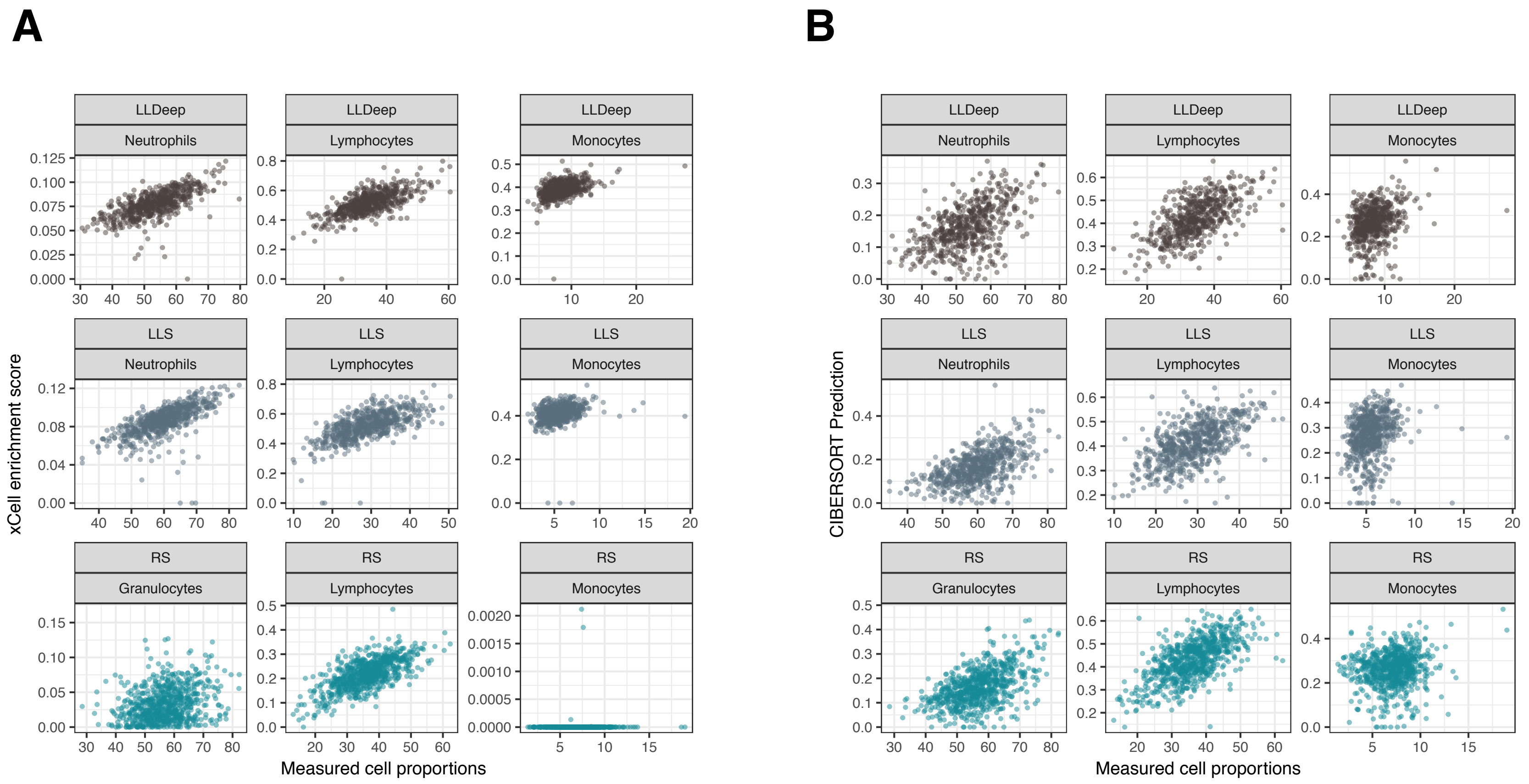

Supp.Figure 5.

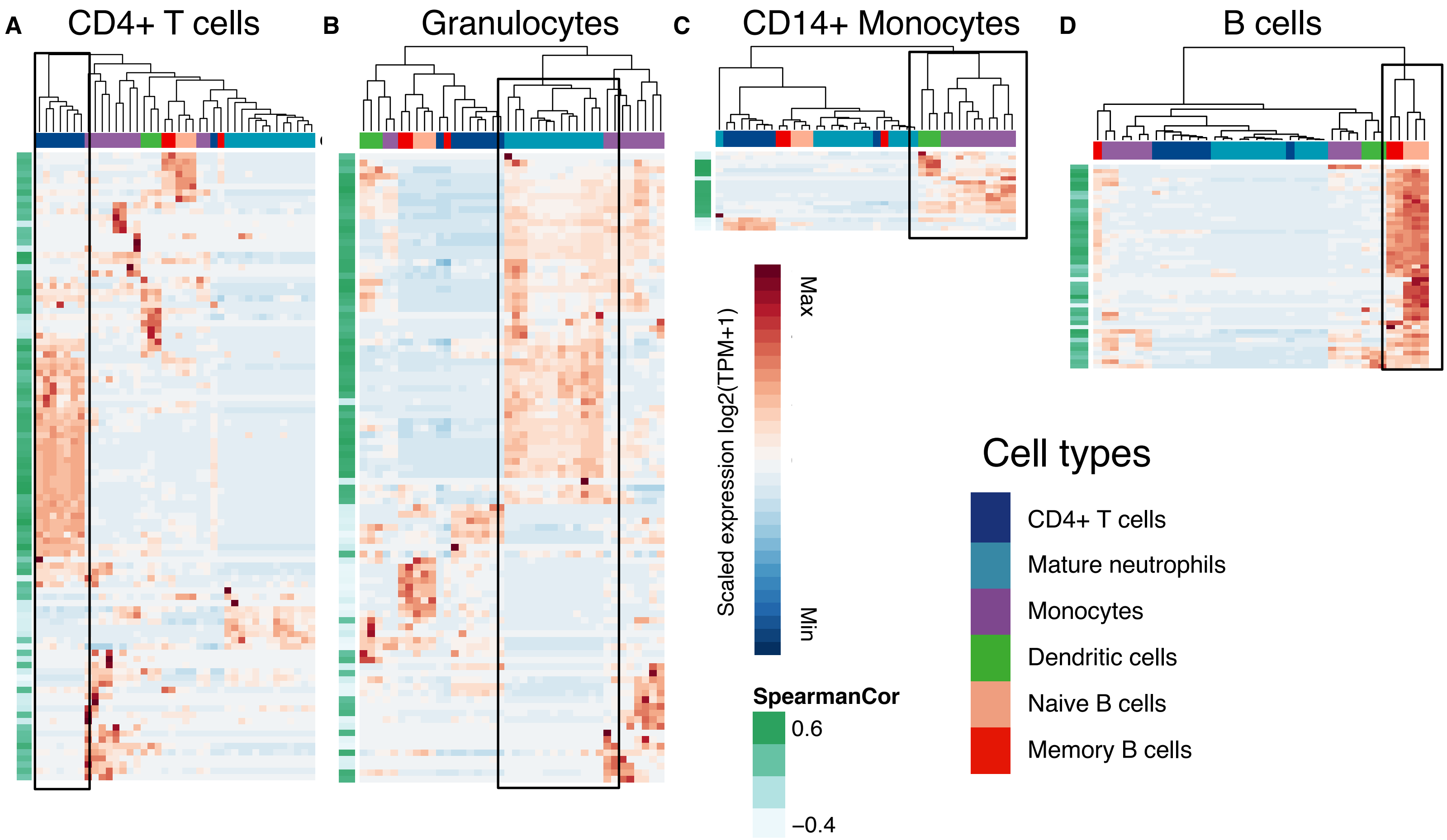

Supp.Figure 6.

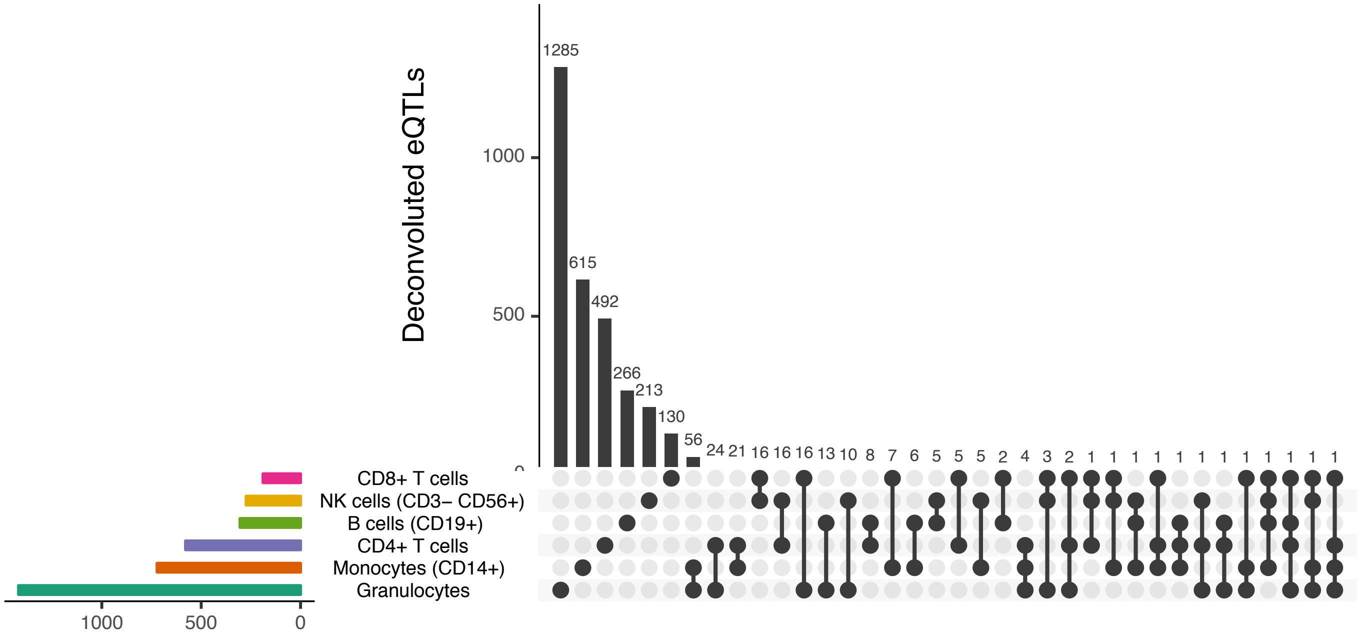

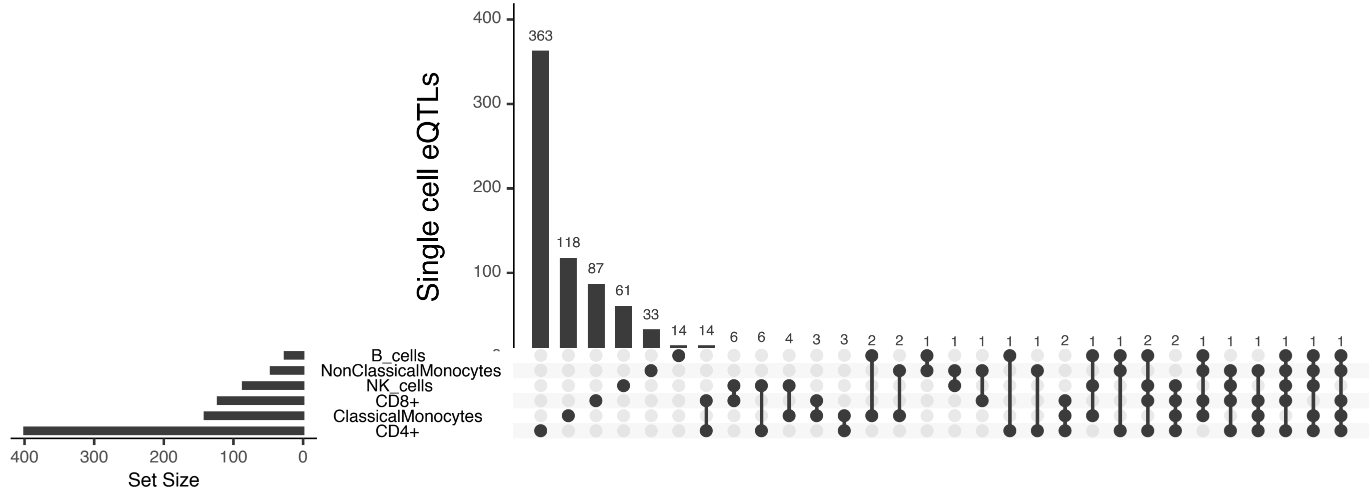

Supp.Figure 7

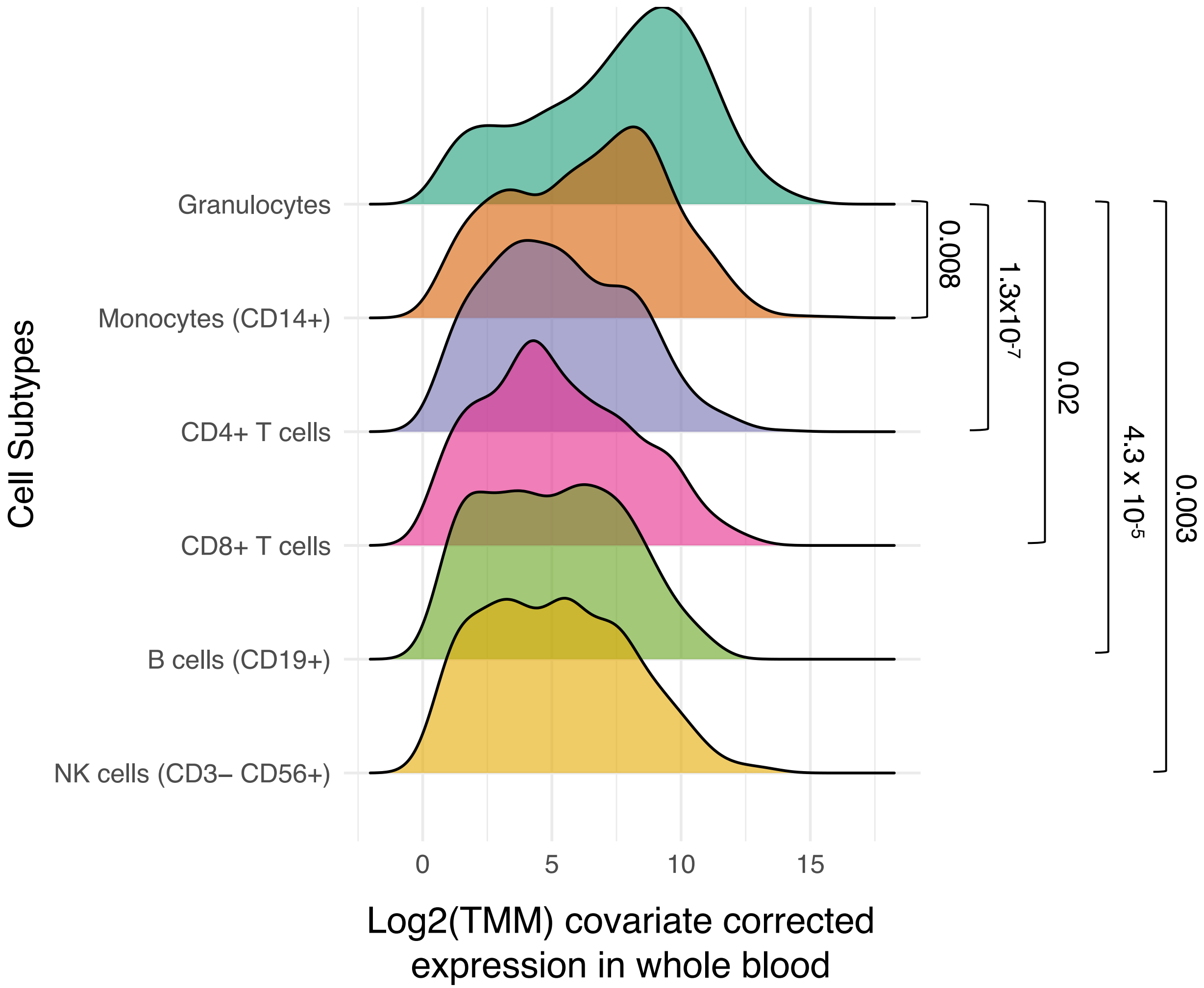

Supp. Figure 8

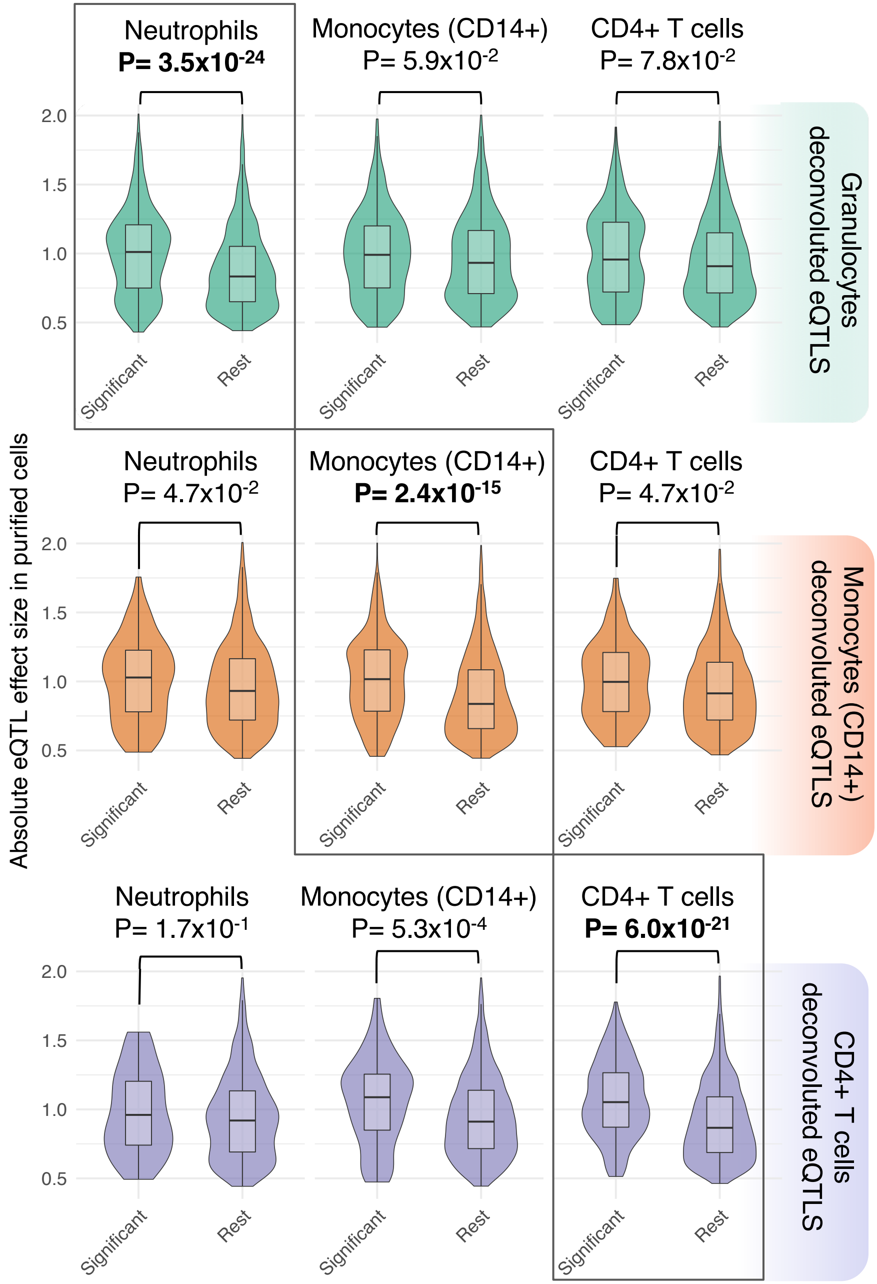

Supp. Figure 9

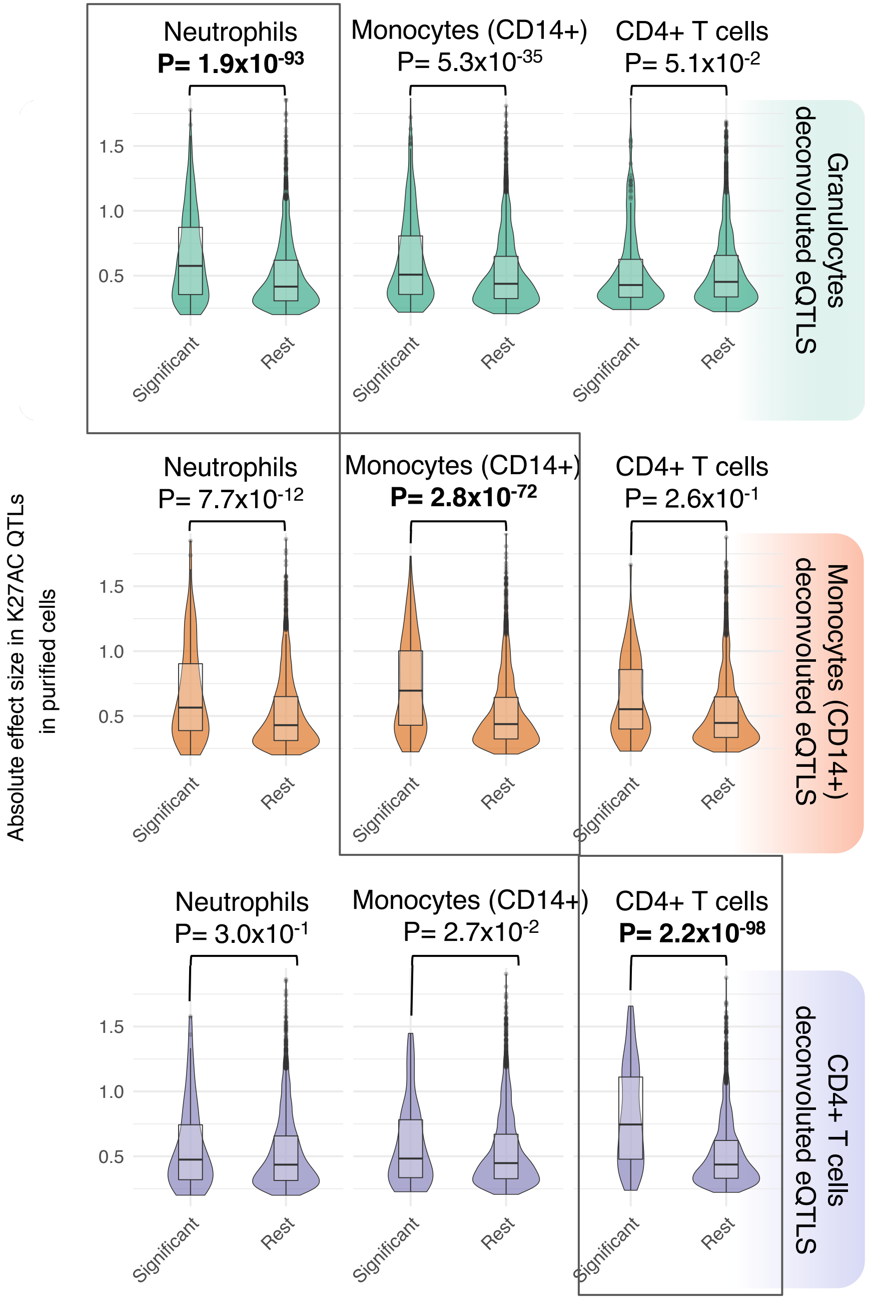

Supp. Figure 10

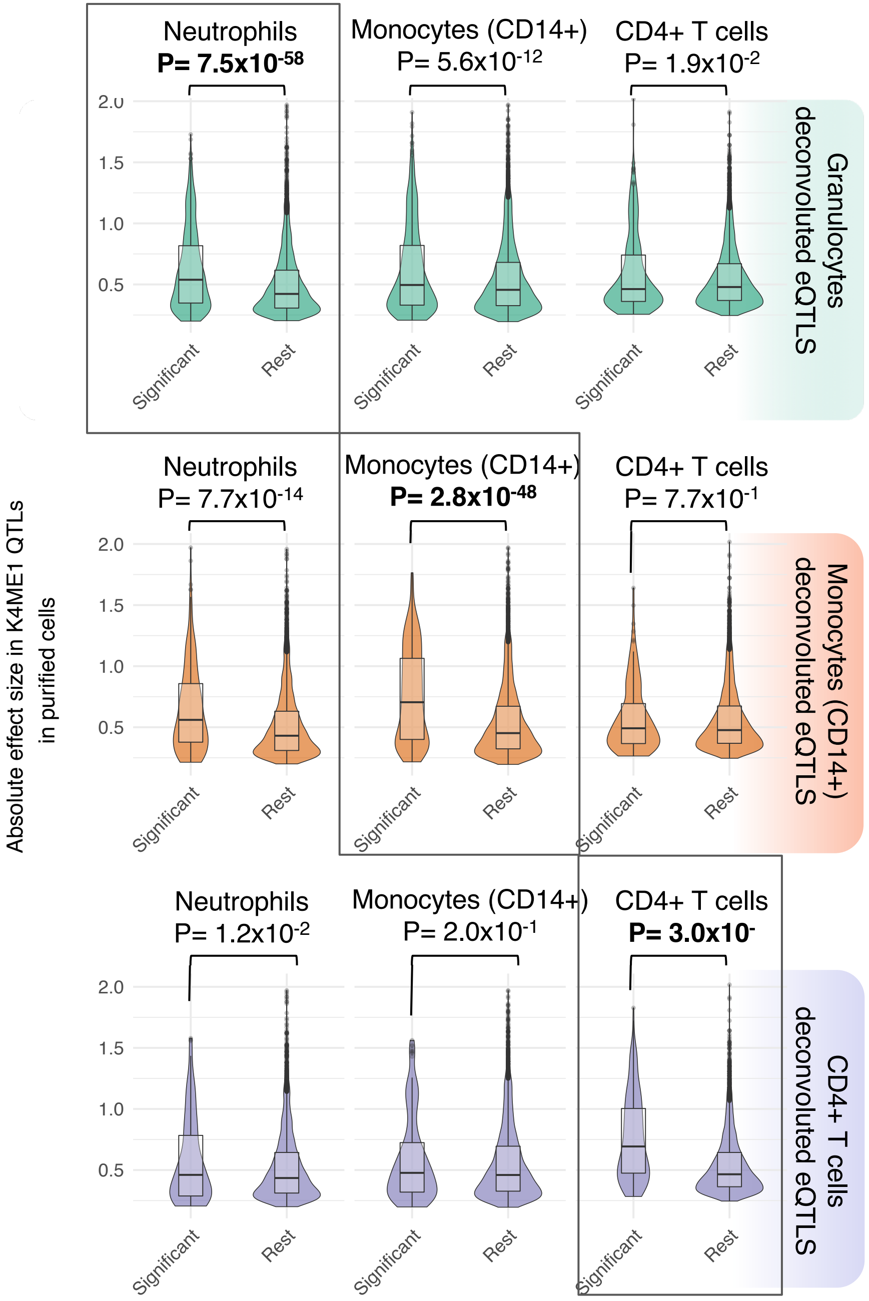

Supp. Figure 11

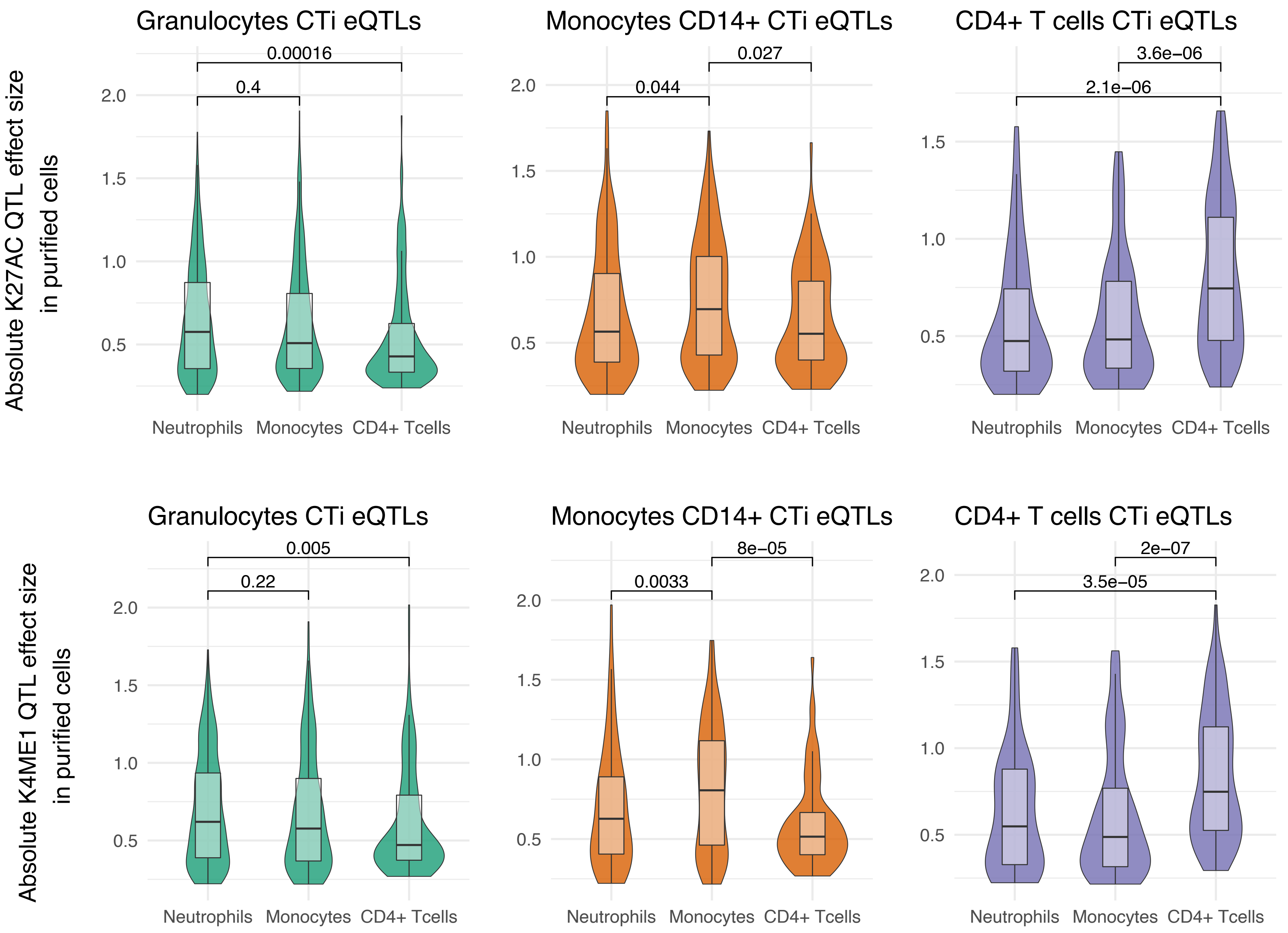

Supp. Figure 12

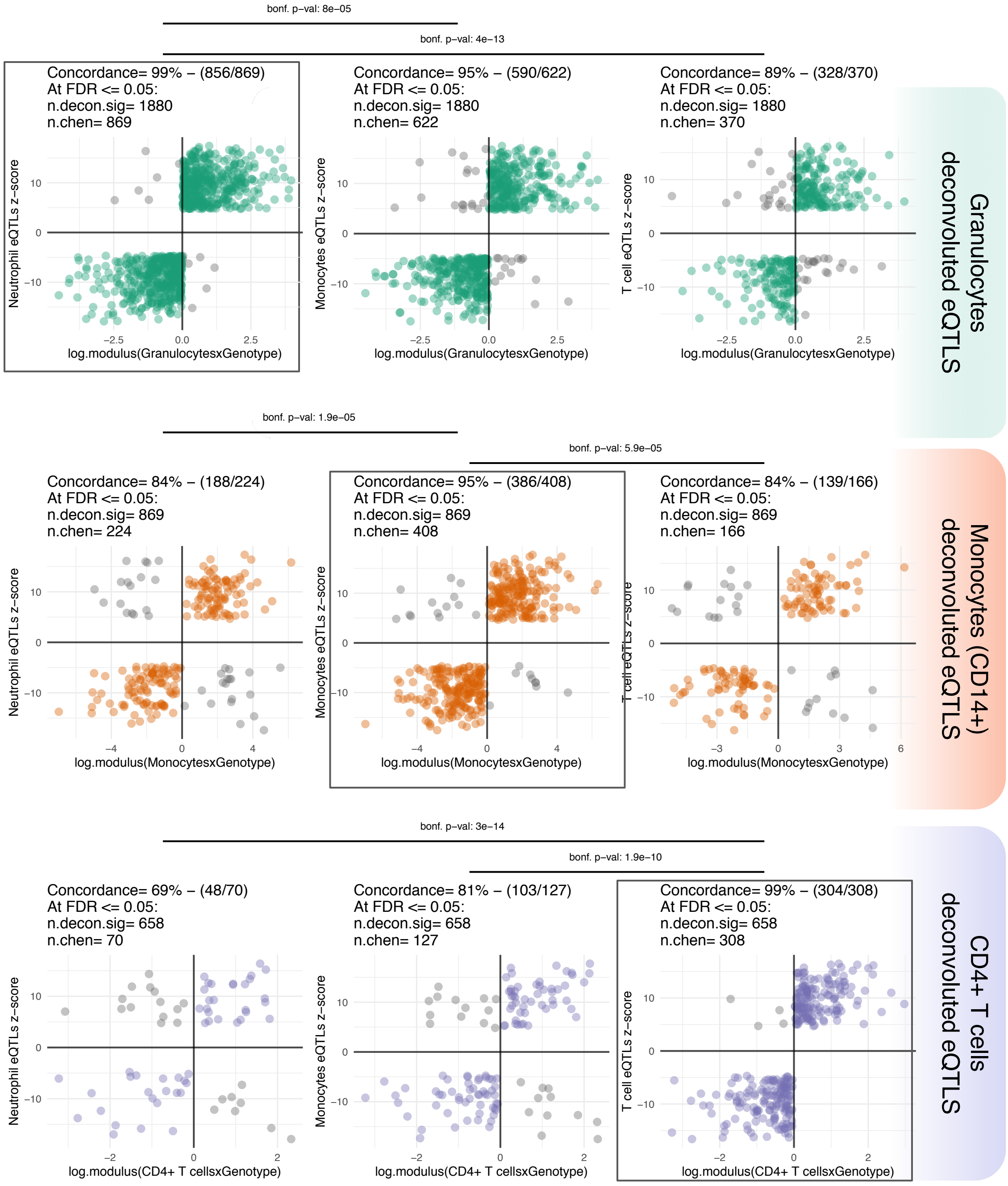

Supp. Figure 13

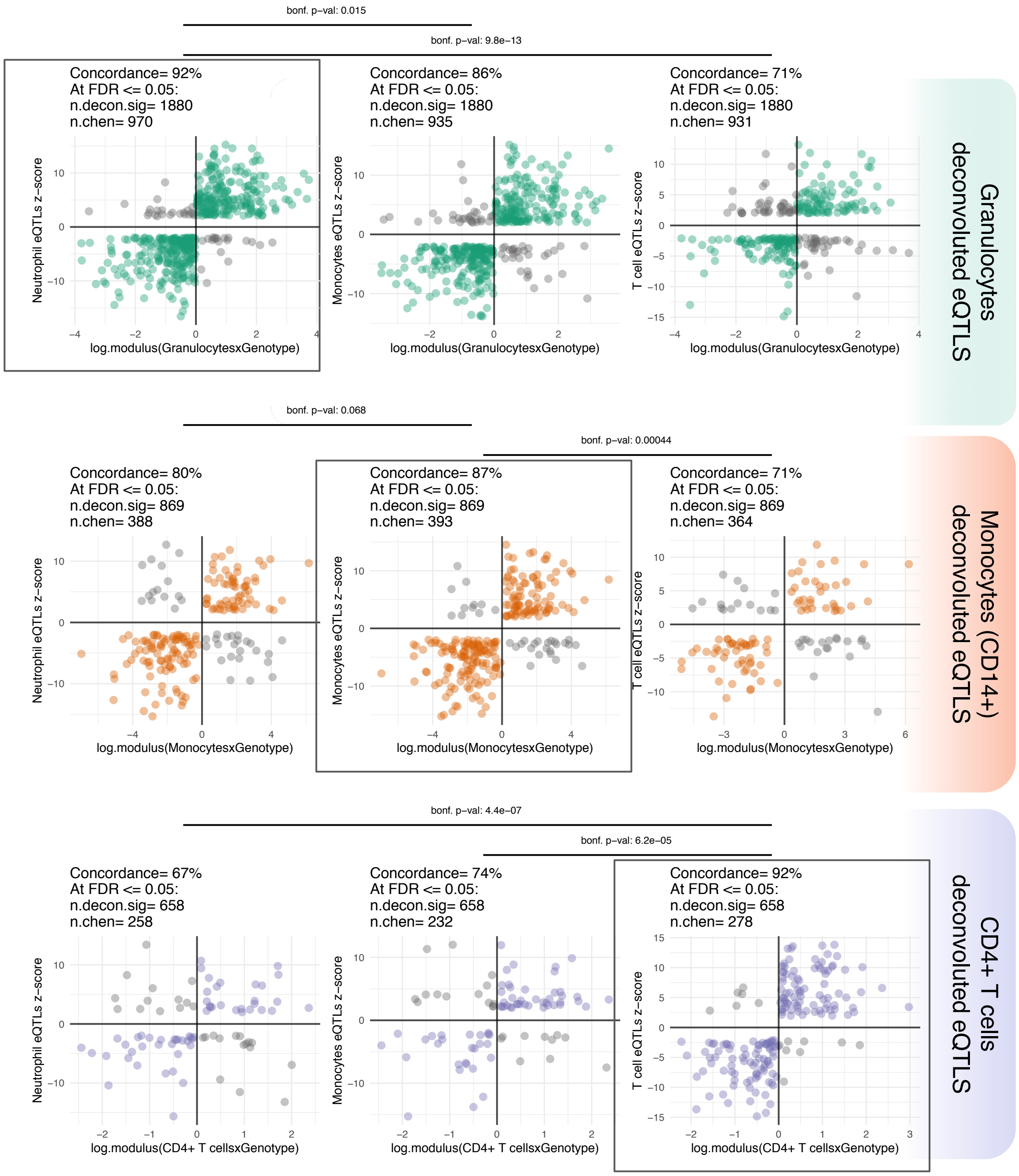

Supp. Figure 14

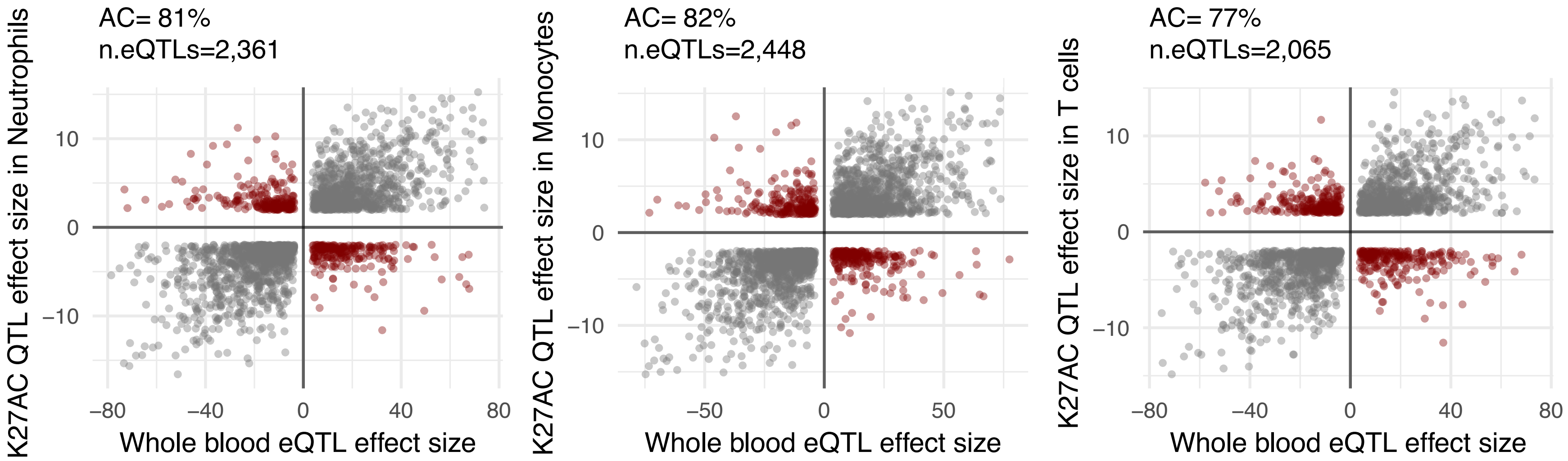

Supp. Figure 15

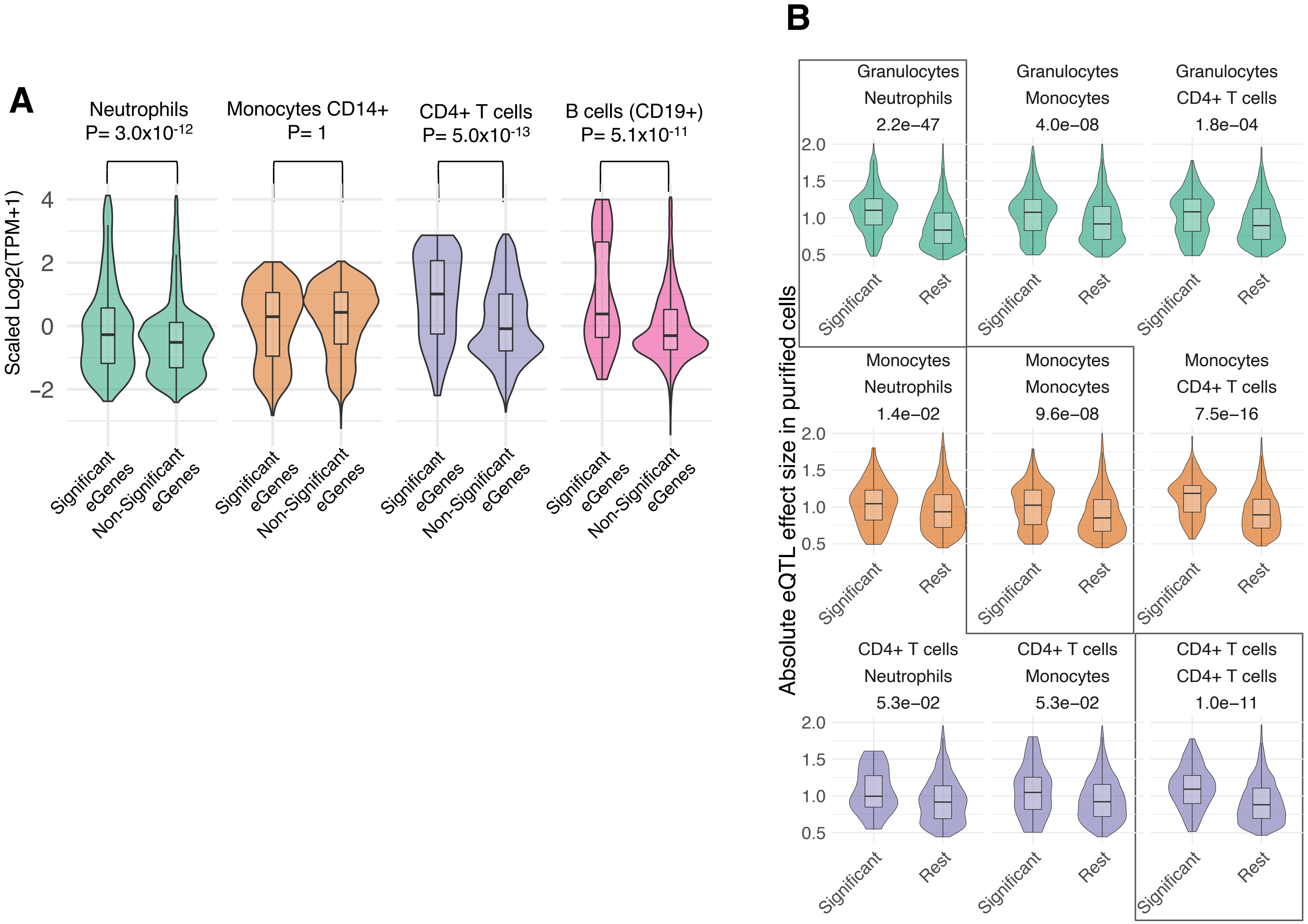

Supp. Figure 16

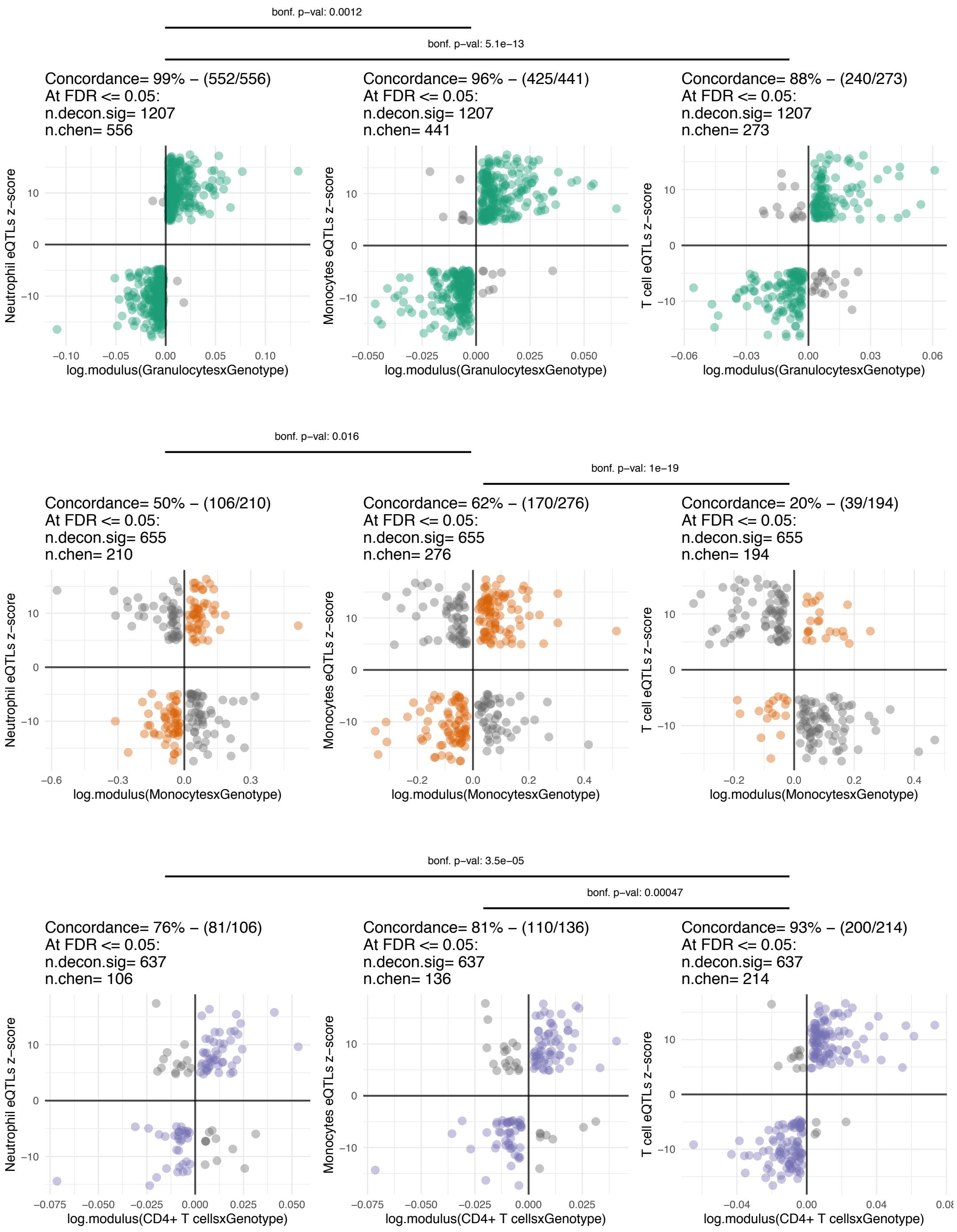

Supp. Figure 17

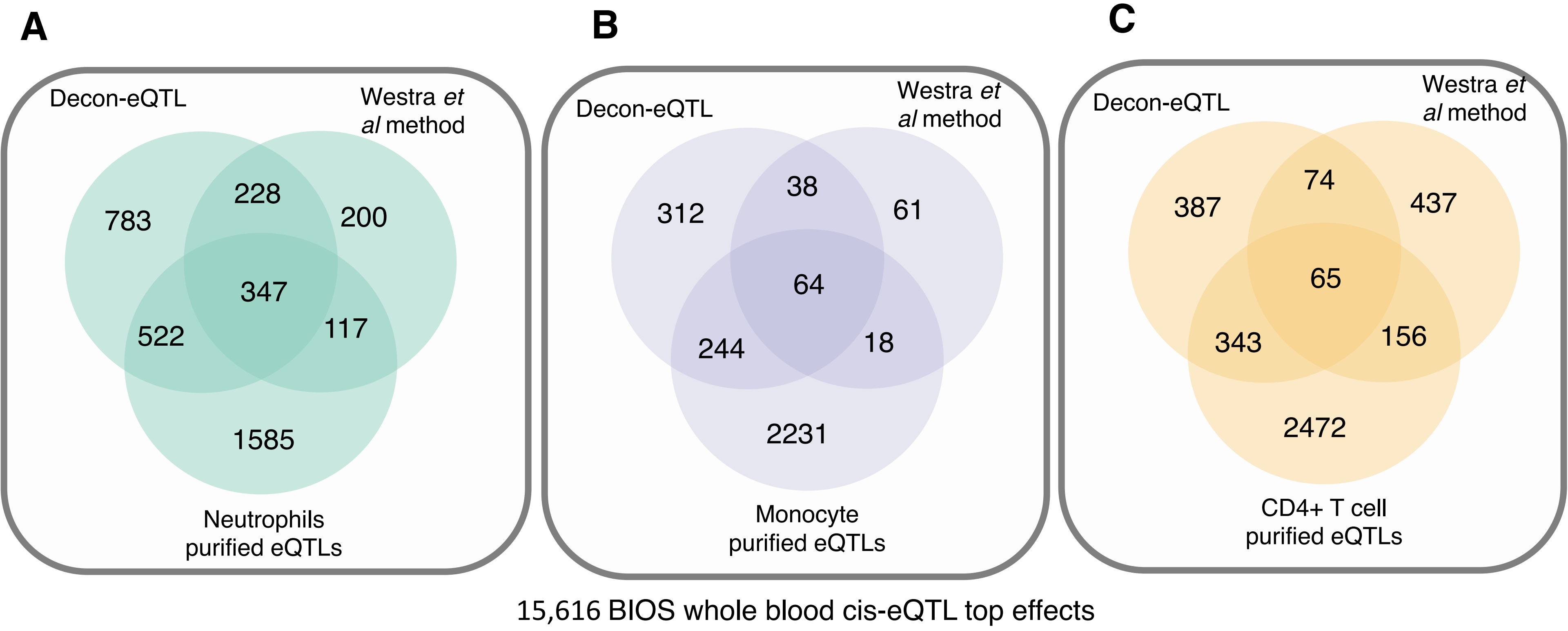

Supp. Figure 18

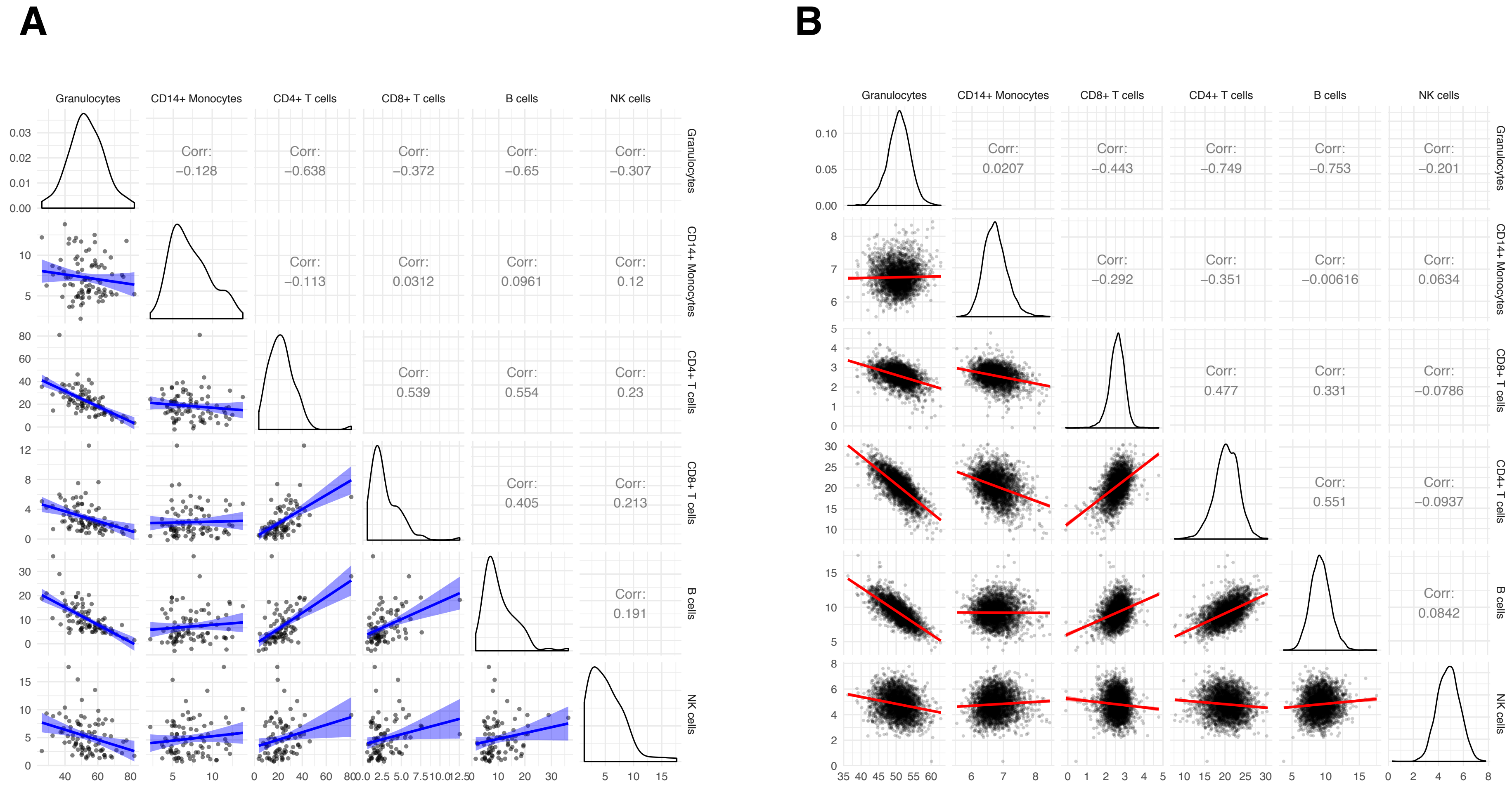

Supp. Figure 19

A

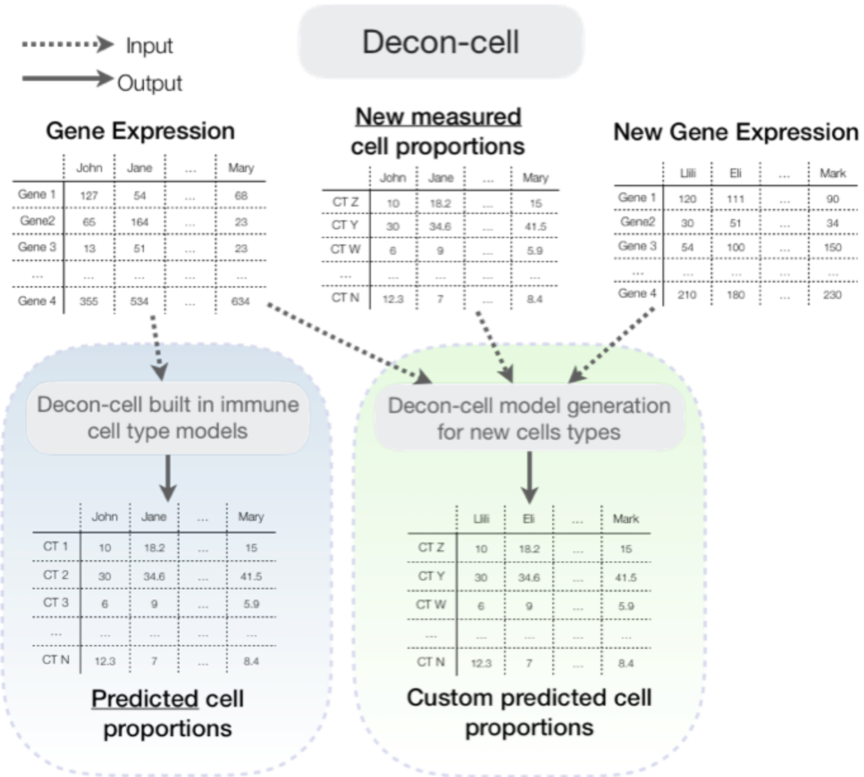

B

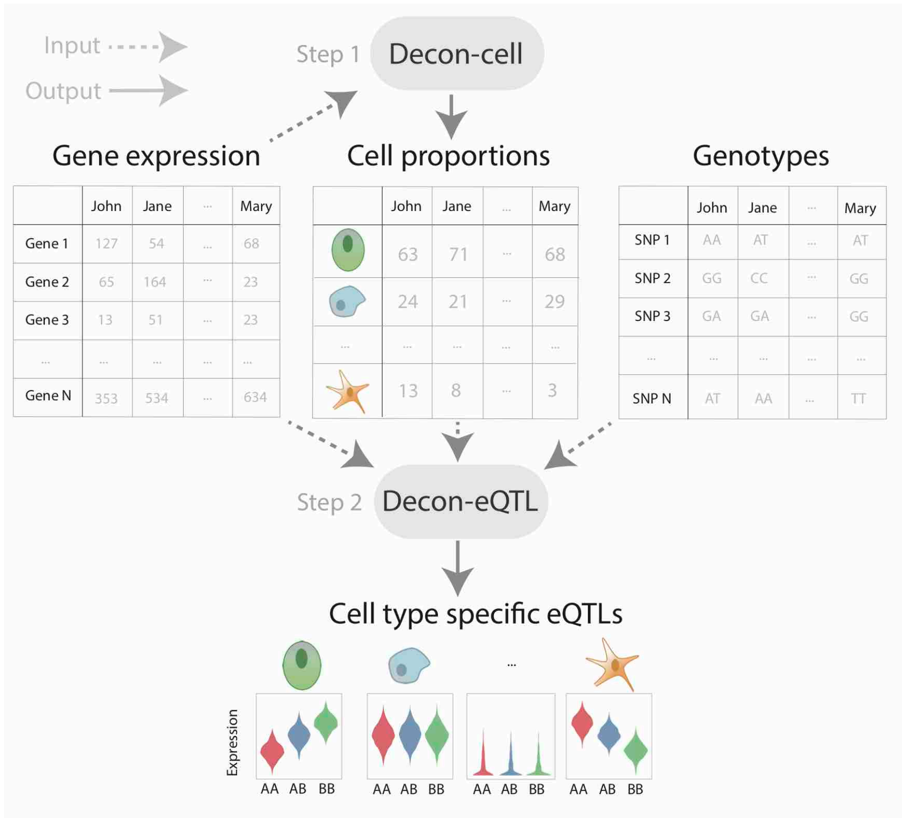

Supplement: Supplementary file 1 — Additional file 1 : Supplementary Figure 1: Prediction performance of Decon-cell within 500FG: The Y-axis represents the 73 immune cell types quantified by FACS in the 500FG cohort. The bar plot on the left panel shows the mean Prediction Performance (Spearman correlation coefficient between predicted and measured cells across 100-fold cross validations). On the right panel, box plots represent the distribution of the Prediction Performance within 100 iterations of the cross validations. A cutoff of mean Prediction Performance ≥0.5 was applied to define predictable cell types (green). Supplementary Figure 2. Signature genes selected for prediction of cell proportions by Decon-cell: (A) Total number of marker genes (genes selected in ≥80% of all models in the 100 iterations) per predictable cell type. Different colors indicate different subpopulations. (B) The number of genes significantly correlated with cell counts (Spearman correlation, adjusted P ≤ 0.05) (y-axis) shows the total number of significantly correlated genes, while the x-axis shows the prediction performance (x-axis). (C) Distributions of the total number of “strongly” correlated genes (absolute Spearman correlation ≥0.3) between predictable and unpredictable cell subpopulations. Supplementary Figure 3. Comparison of prediction performance between Decon-cell and other existing methods. (A) Performance of Decon-cell: the measured (x axis) and predicted cell proportions (y-axis) were compared for neutrophils (given by granulocytes in 500FG), lymphocytes and monocytes CD14+ and granulocytes in three independent cohorts (shown by row, from top to bottom: LLDeep (n = 627); LLS (n = 660); RS (n = 773)). (B) Comparison of prediction performance for Decon-cell, CIBERSORT and xCell in three independent cohorts for a total of 4 major immune subpopulations. Supplementary Figure 4. Prediction performance of xCell and CIBERSORT in three independent Dutch populations (LLDeep, n = 627; LLS, n = 660; RS, n = 773). (A) [file 12859_2020_3576_MOESM1_ESM.pdf]
